# Deconvoluting the T Cell Response to SARS-CoV-2: Specificity Versus Chance and Cognate Cross-Reactivity

**DOI:** 10.3389/fimmu.2021.635942

**Published:** 2021-05-28

**Authors:** Alexander A. Lehmann, Greg A. Kirchenbaum, Ting Zhang, Pedro A. Reche, Paul V. Lehmann

**Affiliations:** ^1^ Research and Development, Cellular Technology Ltd., Shaker Heights, OH, United States; ^2^ Laboratorio de Inmunomedicina & Inmunoinformatica, Departamento de Immunologia & O2, Facultad de Medicina, Universidad Complutense de Madrid, Madrid, Spain

**Keywords:** mega peptide pools, ELISPOT, ImmunoSpot, immune monitoring, COVID-19, T cell affinity

## Abstract

SARS-CoV-2 infection takes a mild or clinically inapparent course in the majority of humans who contract this virus. After such individuals have cleared the virus, only the detection of SARS-CoV-2-specific immunological memory can reveal the exposure, and hopefully the establishment of immune protection. With most viral infections, the presence of specific serum antibodies has provided a reliable biomarker for the exposure to the virus of interest. SARS-CoV-2 infection, however, does not reliably induce a durable antibody response, especially in sub-clinically infected individuals. Consequently, it is plausible for a recently infected individual to yield a false negative result within only a few months after exposure. Immunodiagnostic attention has therefore shifted to studies of specific T cell memory to SARS-CoV-2. Most reports published so far agree that a T cell response is engaged during SARS-CoV-2 infection, but they also state that in 20-81% of SARS-CoV-2-unexposed individuals, T cells respond to SARS-CoV-2 antigens (mega peptide pools), allegedly due to T cell cross-reactivity with Common Cold coronaviruses (CCC), or other antigens. Here we show that, by introducing irrelevant mega peptide pools as negative controls to account for chance cross-reactivity, and by establishing the antigen dose-response characteristic of the T cells, one can clearly discern between cognate T cell memory induced by SARS-CoV-2 infection vs. cross-reactive T cell responses in individuals who have not been infected with SARS-CoV-2.

## Introduction

Traditionally, the assessment of immune memory has relied upon measurements of serum antibodies without queries of the T cell compartment. However, SARS-CoV-2 infection highlights the shortcoming of such a serodiagnostic approach. While the majority of SARS-CoV-2-infected individuals initially develop an antibody response to this virus, false negative results are a concern because not all infected individuals attain high levels of serum antibody reactivity acutely after infection (1–3), and those who do develop detectable antibody reactivity might decline to the limit of detection within a few months (4). In such cases, the detection of T cell memory might be the only evidence of such infection, and is a surrogate of acquired immune protection from SARS-CoV-2 reinfection.

Fueled additionally by evidence that T cell-mediated immunity is required for immune protection against SARS-CoV-2 (5–7), attention has turned to T cell immunodiagnostics trying to establish whether the detection of T cell memory may be a more sensitive and reliable indicator of SARS-CoV-2 exposure than antibodies (8–11). In most studies published so far, SARS-CoV-2-specific memory T cells were detected in the majority of infected individuals, but such were also found in 20-81% of control subjects who clearly could not have been infected by the SARS-CoV-2 virus (9, 12–18). If generalizable, such results would imply that T cell assays are unsuited to reliably identify who has, or has not, been infected by the SARS-CoV-2 virus, providing false positive results in up to 81% of the individuals tested. It should be noted right away, however, that the notion of cross-reactive SARS-CoV-2 antigen recognition by T cells being common in unexposed subjects might be related to the T cell assay itself and the test conditions used, as it was not observed by others (14, 19, 20). Progress with settling the issue of T cell cross-reactivity in SARS-CoV-2 antigen recognition, and identifying suitable test systems, will decide whether T cell diagnostics can reliably detect specific immune memory to SARS-CoV-2 infection/exposure, and possibly identify the immune protected status of those subjects.

Next to possible cross-reactivity, T cell immune diagnostics of SARS-CoV-2 infection faces the challenge of having to reliably detect antigen-specific T cells in blood that occur in very low frequency. The numbers of SARS-CoV-2-specific T cells in blood post-infection is about one tenth of the numbers of T cells specific for viruses that induce strong responses, such as influenza, Epstein Barr (EBV) or human cytomegalovirus (HCMV) (11, 21), and reliably detecting even the latter is at the border of current technology. Possibly further complicating matters, the frequencies of SARS-CoV-2-specific T cells are even lower in subjects who underwent a mild or asymptomatic SARS-CoV-2 infection as compared to those who developed more severe COVID-19 (12, 20, 22, 23), but this notion has not been supported by others (5, 24). Owing to these low T cell frequencies, and the antigen-induced signal being small in magnitude, any contribution of cross-reactive T cell stimulation will interfere with the reliable detection of genuine SARS-CoV-2-specific T cells. Setting up clear cut-off criteria for identifying antigen-specific T cell memory is therefore paramount.

Because T cell assays rely upon detecting SARS-CoV-2 antigen-specific T cells in blood *via* memory T cell re-activation *ex vivo*, the choice and formulation of the SARS-CoV-2 antigen itself used for the T cell recall will critically define the assay result. As the epitope utilization in the T cell response to SARS-CoV-2 is not yet known, by necessity, the aforementioned T cell diagnostic efforts tailored toward this virus have relied either on pools of hundreds of peptides that cover the entire proteome of the virus, or on pools of a multitude of predicted epitopes (mega peptide pools). Traditional T cell immune monitoring efforts, however, have called for the utilization of select, highly purified individual peptides whose specificity has been carefully established. Presently it is unproven whether pools of hundreds of unpurified peptides are even suited for reliable T cell diagnostics, and whether false positive or false negative results obtained using them are inherent to the recall antigen formulation. The chance for T cell cross-reactivity can be expected to increase with every peptide added into a pool, multiplying the chance for false positive results. Conversely, irrelevant peptides (those not recognized by T cells) also present in the pool can be expected to compete with the actually recognized T cell epitopes for binding to HLA molecules, possibly causing false negative results (25). To our knowledge, it has not yet been systematically addressed whether and how chance cross-reactivity or peptide competition affects T cell immune monitoring results when mega peptide pools are used for testing. Instead of relying on third party mega peptide pools as the proper negative control to establish the background noise of the T cell assay, in all SARS-CoV-2 studies published so far, the mega peptide pool-induced T cell activation has been compared to PBMC cultured in media alone, in the absence of any exogenously added peptide. In this report we introduce suitable negative control mega peptide pools, and using them, we address how to reliably detect even the very low frequency SARS-CoV-2 antigen-specific T cells in subjects who have undergone mild SARS-CoV-2 infection.

Cognate T cell cross-reactivity between related pathogens, such as SARS-CoV-2 and seasonal common cold coronaviruses (CCC), needs to be distinguished from the aforementioned chance cross-reactivities. In cognate cross-reactivity, the TCR binds peptide sequences of two antigens that have sequence similarities. In the majority of documented cases, such cross-reactive peptide sequences differ in only one or two amino acids with an additional requirement being that the exchange of amino acid(s) does not interfere with the peptides’ binding to, and folding in, the peptide binding groove of the restricting HLA molecule. Examples of experimentally verified cognate cross-reactivities include T cell recognition of serotypes of the Dengue virus (26, 27), influenza A virus strains (28–31), hepatitis C virus escape variants (32), and HIV epitope variants from different clades (33, 34).

However, it remains controversial how exclusively specific T cell recognition is in general (35). On one hand, there are reports suggesting that T cell recognition might be highly promiscuous with individual T cell clones being able to cross-reactively recognize 10^6^ different peptides (36). On the other hand, changing even a single amino acid in the presented peptide frequently abrogates T cell recognition, in particular if the change affects the binding of the peptide for the restricting MHC molecule, its conformation when bound to the MHC molecule, or when involving a TCR contact residue. While some studies have indicated an extremely low frequency of T cell cross-reactions between unrelated peptides (37–39), other studies (relying on tetramers) claim the opposite (40, 41). Accordingly, it needed to be addressed what impact TCR chance cross-reactivity has on *ex vivo* T cell monitoring when using mega peptide pools in general, and for SARS-CoV-2 antigen recognition in particular.

When T cell activation was seen in SARS-CoV-2-unexposed individuals using SARS-CoV-2 mega peptide pools for recall, the finding was interpreted as cognate cross-reactivity with related coronaviruses that cause harmless, common cold-like epidemies in the human population (42). There are four seasonal coronavirus strains, 229E, NL63, OC43, and HKU1, which cause pandemics in multiyear infection cycles in the human population world-wide (43). Although in any given year only 15-30% of humans displaying symptoms of common cold are indeed infected by one of these seasonal coronaviruses, 90% of the adult human population eventually becomes seropositive for at least three of these coronaviruses (44–46). From the perspective of T cell immune diagnostics of SARS-CoV-2, such cross-reactive T cell responses would generate false positive results. Another major scope of the present study was to establish to what extent cognate T cell cross-reactions of seasonal coronavirus antigens interferes with the detection of T cell memory induced by the SARS-CoV-2 virus itself.

The SARS-CoV-2 pandemic has made its rounds for nearly a year by now, yet its prevalence in the human population remains unknown as most of those infected go undiagnosed, having developed mild or no clinical symptoms at all (47). By now serum antibodies may no longer be reliable in revealing, in retrospect, who has or has not been infected more than 3 months ago. If measurements of T cell memory would also fail to provide this information, our understanding of SARS-CoV-2’s prevalence would remain shrouded. Should vaccines under present development fail, without this information, it will remain guesswork to decide whether and when sufficient herd immunity has developed in a population, or if robust immunity develops at all following natural infection (48). Without knowing who has or has not been infected by SARS-CoV-2, one cannot distinguish whether a candidate vaccine can prime a protective immune response in naïve individuals, or whether it merely boosts immunity that has been pre-established by the natural infection. Without this information, all those individuals – possibly the majority of the population - who already went through an uncomplicated SARS-CoV-2 infection and might be protected from re-infection, or are prone to develop a mild disease if reinfected again, need to continue to live in fear of contracting a potentially lethal disease.

In this report we sought solutions to deconvolute T cell reactivity to SARS-CoV-2 mega peptide pools so as to clearly distinguish between individuals who have or have not been infected with this virus.

## Materials And Methods

### Peripheral Blood Mononuclear Cells

Pre-COVID Era Donors. PBMC from healthy human donors were obtained from CTL’s ePBMC library (CTL, Shaker Heights, OH, USA) collected prior to Dec 31, 2019. The PBMC were collected in FDA-registered collection centers from IRB-consented healthy human donors by leukapheresis using the Spectra Optia^®^ Apheresis System CMNC collection protocol using ACD-A as the anticoagulant. All PBMC were from healthy adults who had not taken medication within a month of the blood draw that might influence their T cell response. In addition, tests were done on each donor at the collection centers’ CLIA-certified laboratories to identify common infections, including Human Immunodeficiency Virus (HIV). Subjects positive for HIV were disqualified from the ePBMC library. The donors’ age, sex, and ethnicity are shown in **Supplementary Table 1**. The cryopreservation procedure used fully preserves the thawed PBMC’s functionality in T cell assays when compared to the freshly isolated PBMC (49–51).

SARS-CoV-2-Infected Donors. PBMC of subjects were collected under Advarra Approved IRB #Pro00043178, CTL study number: GL20-16 entitled COVID-19 Immune Response Evaluation. All subjects tested positive for SARS-CoV-2 RNA in PCR performed on nasal swabs, and these PCR tests were performed in accredited medical laboratories. All such donors underwent mild COVID infection, from which the subjects fully recovered within one or two weeks. These subjects were bled between 2 weeks and 3 months post recovery (median of 24 days). The donors’ age, sex, and ethnicity are shown in **Supplementary Table 1**. SARS-CoV-2 antigen-specific serum antibodies were evaluated for this cohort in parallel with Pre-COVID Era donors to additionally verify their infection (**Supplementary Figure 1**).

The cryopreserved cells were thawed following an optimized protocol (51) resulting in viability exceeding 90% for all samples. The PBMC were resuspended in CTL-Test™ Medium (from CTL). CTL-Test™ Medium is serum-free and has been developed for low background and high signal performance in ELISPOT assays. The number of PBMC plated in the ELISPOT experiments was 2 x 10^5^ viable PBMC per well.

### ELISA Assays

MaxiSorp 96-well microplates (Thermo Fisher) were coated with recombinant SARS-CoV-2 Nucleocapsid (RayBiotech, Peachtree Corners, GA), truncated Spike protein (S1 domain) (The Native Antigen Company, Oxford, UK) or receptor binding domain (RBD) (Center for Vaccines and Immunology (CVI), UGA, Athens, GA) at 2µg/mL in PBS overnight at 4°C. Plates were then blocked with ELISA blocking buffer containing 2% w/v bovine serum albumin in PBS with 0.1% v/v Tween20 (PBS-T) (Sigma-Aldrich) for 1 h at room temperature. Donor plasma were serially diluted in assay plates and incubated overnight at 4°C. Plates were then washed with PBS prior to addition of horseradish peroxidase-conjugated anti-human IgG detection reagents (from CTL) and incubation for 2 h at room temperature. Plates were then washed with PBS prior to development with TMB chromogen solution (Thermo Fisher). 1M HCl was used to stop conversion of TMB and optical density was measured at 450nm (OD_450_) and 540nm (OD_540_) using a Spectra Max 190 plate reader (Molecular Devices, San Jose, CA USA). Optical imperfections in assay plates were corrected through subtraction of OD_540_ values. Antigen-specific IgG concentrations are reported as μg/mL IgG equivalents and were interpolated from a standard curve generated using an IgG reference protein (Athens Research and Technology, Athens, GA) coated directly into designated wells of assay plates.

### Antigens and Peptides

#### Mega Peptide Pools

Selecting the right peptides is critical for the detection of *in vivo* primed T cells. In general, T cell immune monitoring has relied on three fundamentally different approaches to accomplish this goal (reviewed in 64). The first approach relies upon peptides that have been experimentally verified as T cell epitopes of an antigen. Such information is scarce for SARS-CoV-2, as it has only recently piqued the interest of the immune monitoring community. In the second approach, there is the option to attempt to predict epitopes *in silico*. The group of A. Sette has been pioneering this approach for SARS-CoV-2 (13). While HLA binding of peptides can be accurately predicted, this does not necessarily predict the immune dominance of such peptides (52). The third approach, which we selected for this study, relies upon the agnostic use of peptides. Here, peptide libraries are created with the individual peptides, 15 amino acids long, systematically covering the amino acid sequence of the antigen of interest. Such peptides are pooled for each antigen resulting in the mega peptide pools of SARS-CoV-2 as defined in more detail in **Supplementary Table 2**.

All mega peptide pools used in this study are products of, and were purchased from JPT (Berlin, Germany). The peptide pools representing the individual antigens are shown in **Supplementary Table 2**. All these mega peptide pools consisted of 15-mer peptides that covered the entire amino acid (aa) sequence of the respective proteins in steps (gaps of) 4 aa. All mega peptide pools were tested at a final concentration of 1.5 µg/mL of each peptide within the pool at the highest concentration, followed by three 1 + 2 (vol + vol) serial dilutions, as specified in the Tables. All mega peptide pools were delivered as lyophilized powder. The individual peptide pools were initially dissolved following the manufacturer’s directions in 40 µl DMSO, followed by addition of 210 µl of PBS generating a “primary peptide stock solution” at 100 µg/mL (0.1mg/mL) with 16% v/v DMSO. From each of these wells, a “secondary peptide stock solution” was prepared in a 96-Well deep well plate, with peptides starting at 3 µg/mL which were then threefold serially diluted. Using a 96-well multichannel pipettor, 100µl was transferred “en block” into pre-coated ImmunoSpot^®^ assay plates. Finally, 100 µL of PBMC (containing 2 x 10^5^ cells) in CTL-Test™ media was added “en block” to achieve the desired final peptide concentrations of 1.5, 0.5, 0.17 and 0.06 µg/mL in the ELISPOT assay. The final concentration of DMSO in the ELISPOT assay at 1.5 µg/ml of peptide was therefore 0.24% vol/vol, a concentration at which DMSO does not interfere with the test result (see **Supplementary Figure 2**).

#### Positive Controls

CEFX, by JPT Peptide Technologies, Berlin, Germany (Product Code: PM-CEFX) is a pool of 176 known peptide epitopes for a broad range of HLA subtypes – class I and class II – and different infectious agents, namely *Clostridium tetani*, Coxsackievirus B4, *Haemophilus influenza*, *Helicobacter pylori*, Human adenovirus 5, Human herpesvirus 1, Human herpesvirus 2, Human herpesvirus 3, Human herpesvirus 4, Human herpesvirus 5, Human herpesvirus 6, Human papillomavirus, JC polyomavirus, Measles virus, Rubella virus, *Toxoplasma gondii*, and Vaccinia virus. These peptides are 9-15 amino acids long and have been selected to recall both CD4+ and CD8+ T cells. CEFX was tested at 1 μg/mL.

CPI: protein antigens of CMV, Parainfluenza and Influenza viruses. CPI was from and is available through CTL, Catalog #CTL-CPI-001. CPI was tested at 6.25 µg/mL.

CERI: 124 peptides of CMV, EBV, RSV, and Influenza virus. The individual peptides, 9 amino acids long, were selected based on peptide binding predictions for a broad range of HLA class I alleles expressed in all human races, and diverse ethnic subpopulations. CERI was from and is available through CTL, Catalog # CTL-CERI-300. CERI was tested at 1 μg/mL.

All three positive controls have been introduced recently (53) as a superior alternative to the CEF peptide pool.

### Human IFN-γ ELISPOT Assays

Single-color enzymatic ImmunoSpot^®^ kits from CTL were used for the detection of *in vivo*-primed IFN-γ- producing Th1 type memory T cells. Test procedures followed the manufacturer’s recommendations. In brief, peptides were plated at the specified concentrations into capture antibody-precoated ELISPOT assay plates in a volume of 100 µL per well, dissolved in CTL-Test™ Media. The plates with the antigen were stored at 37°C in a CO_2_ incubator for less than an hour until the freshly thawed PBMC were ready for plating. The PBMC were added at 200,000 viable cells/well in 100 µL CTL-Test™ Media and cultured with the peptides for 24h at 37°C and 9% CO_2_ in an incubator. After removal of the cells, addition of detection antibody, and enzymatic visualization of plate-bound cytokine, the plates were air-dried prior to scanning and counting of spot forming units (SFU). ELISPOT plates were analyzed using an ImmunoSpot^®^ S6 Ultimate Reader, by CTL. SFU numbers were automatically calculated by the ImmunoSpot^®^ Software for each stimulation condition using the Autogate™ function of the ImmunoSpot^®^ Software that enables scientifically validated, objective counting (54). Stringent gating to attain low background inherently reduces the antigen-induced spot count, but increases the signal to noise performance of the ELISPOT test. Occasional subjects have an elevated background that results from increased IFN-γ production by cells of the innate immune system due to underlying cellular activity in such subjects at the time of the blood draw (55). The above statistics-based gating and analysis approach is suited to dissect the antigen-triggered T cell signal from the respective background.

### Statistical Analysis

As ELISPOT counts follow Gaussian (normal) distribution among replicate wells (56), the use of parametric statistics was justified to identify positive and negative responses, respectively. Positive responses were defined as SFU counts exceeding 3 SD of the mean SFU counts of the specified negative control, identifying such at 99.7% confidence.

## Results And Discussion

### Experimental Design for Assessment of T Cell Memory to SARS-CoV-2

#### The Rationale for Selecting IFN-γ ELISPOT for Detecting SARS-CoV-2-Specific Memory T cells

T cell immune monitoring aims at detecting *in vivo* expanded and differentiated antigen-specific T cell populations directly *ex vivo*, either in freshly isolated PBMC, or in PBMC that have been cryopreserved following protocols that maintain full T cell functionality upon thawing the cells (50). The number (frequency) and functions (e.g. cytokine signature) of antigen/peptide-specific T cells need to be measured as present in the body without inducing additional clonal expansions or T cell differentiation *in vitro* during the short-term *ex vivo* antigen stimulation that is required to detect the antigen-reactive T cells. In ELISPOT/ImmunoSpot^®^ assays, antigen- (peptide)-specific T cells present in the PBMC become activated, and start producing cytokine. This cytokine is captured on a membrane around each secreting T cell, resulting in a cytokine spot (a spot forming unit, SFU). Counting of SFUs permits to establish, at single-cell resolution, the number of antigen-triggered cytokine-producing T cells (55), and thus the frequency of such cells in PBMC. In this study we focused on IFN-γ measurements because, in subjects who successfully overcome SARS-CoV-2 infection, Th1 cells have been reported to prevail by far (5, 9, 18, 57, 58). Th1 cells have been implicated as a protective class of response while Th2 and Th17 have been linked to immune pathology (57, 59). Furthermore, the standard 24h IFN-γ ELISPOT assay detects *in vivo*-primed Th1 effector memory cells only; naïve T cells or central memory cells are not detected in this assay as the latter require several days of differentiation following antigen encounter before they begin secreting IFN-γ (60, 61).

We also selected the *ex vivo* ELISPOT platform because it requires as few as 200,000 peripheral blood mononuclear cells (PBMC) per antigen stimulation condition, and this assay lends itself to high-throughput analysis. Utilizing only 32 million PBMC per subject (obtainable from 32 mL of blood), we established 155 T cell reactivity datapoints per subject, testing 37 mega peptide pools (see **Supplementary Table 2**), each at four concentrations, plus 4 media and 3 positive control wells, all in a single high-throughput experiment. In the mega peptide pools, the individual peptides were present at 1.5 µg/mL at the highest concentration tested, and in 0.5 µg/mL, 0.17 µg/mL, and 0.06 µg/mL in the subsequent 1 + 2 (vol + vol, 3-fold) serial dilutions. Instead of using replicates, these serial antigen dilutions served not only to confirm positive results, but additionally permitted us to establish the affinity/avidity of the responding T cells. Distinguishing between high and low affinity SARS-CoV-2-specific T cells might help shed light on T cell immunity operational in individuals undergoing alternative outcomes following infection (62).

#### The Rationale for Using Mega Peptide Pools for Detecting SARS-CoV-2-Specific Memory T cells

Due to the highly individualized nature of T cell epitope recognition in general, for which evidence is also starting to accumulate for SARS-CoV-2 (11–13, 16, 20, 58), and due to the size of the virus whose genome is approximately 29.8 kb (63), there are only two viable options for selecting peptides for a comprehensive assessment of T cell immunity to SARS-CoV-2. One option is to perform *in silico* epitope predictions, and such has already been reported for SARS-CoV-2 (64), but their accuracy has recently been convincingly called into question (52, 65). Moreover, for a comprehensive assessment, the epitope predictions would need to be customized for each test subject, accounting for all HLA class I and class II molecules expressed in each individual. Because it is impractical to individualize predicted peptide epitopes for each subject, we have elected to take the agnostic route, in which the entire sequence of each protein antigen is covered by a series of overlapping peptides. However, this means that, dependent upon the length of the protein, hundreds of peptides need to be combined into mega pools (see **Supplementary Table 2**). While, in theory, the mega peptide pool approach permits systematic coverage of all possible T cell epitopes within a virus, it introduces an as yet undefined dimension: chance cross-reactivity between unrelated peptides.

We tested 15-mer peptides which, based upon their length, preferentially stimulate CD4+ T cells but *via* cross-presentation also recall antigen-specific CD8+ T cells (66). The activation of T cells *vs.* cells of the innate immune system is internally controlled in such recall assays using unpurified PBMC because possible contaminations of peptides, e.g. with LPS, would trigger IFN-γ production in all subjects irrespective of their immune memory status.

#### The Rationale for Selecting Negative Control Mega Peptide Pools

To account for chance T cell cross-reactivity, we tested mega peptide pools covering foreign antigens to which it is unlikely that the test subjects have been exposed (one Ebola virus peptide pool, five HIV antigen pools) and a self-antigen, Actin, that due to its abundance in the body is likely to have established self-tolerance. These mega peptide pools are defined in **Supplementary Table 2**, including the number of peptides contained in each.

#### Avoiding Inter-Assay Variations

To reduce assay variables, all peptide pools used in this study were from the same vendor, were synthesized, stored, dissolved and tested the same way, and had the exact same formulation consisting of 15 amino acid-long peptides that systematically walk the entire sequence of the respective proteins in steps of 11 amino acids. Taking advantage of the high-throughput suitability of ELISPOT, all the peptide pools (**Supplementary Table 2**) and their dilutions were tested on each PBMC donor in a single experiment, which rendered the peptides the only assay-dependent variable. This approach therefore permitted us to firmly establish within each PBMC sample the number of Th1 T cells responding to the different mega peptide pools, and thus to compare the frequencies of the respective mega peptide pool-reactive T cells within each PBMC donor, and among donors in the cohorts.

We compared T cell reactivity to all the above mega peptide pools in 18 healthy Pre-COVID-19 Era Subjects and in the 9 individuals who recovered from mild SARS-CoV-2 infection as verified by PCR. In the following we describe and interpret the results.

### Classic Single Antigen and Single Antigen Dose-Based Data Analysis Does Not Permit to Distinguish Between SARS-CoV-2-Infected Subjects and Controls

**Figure 1:**
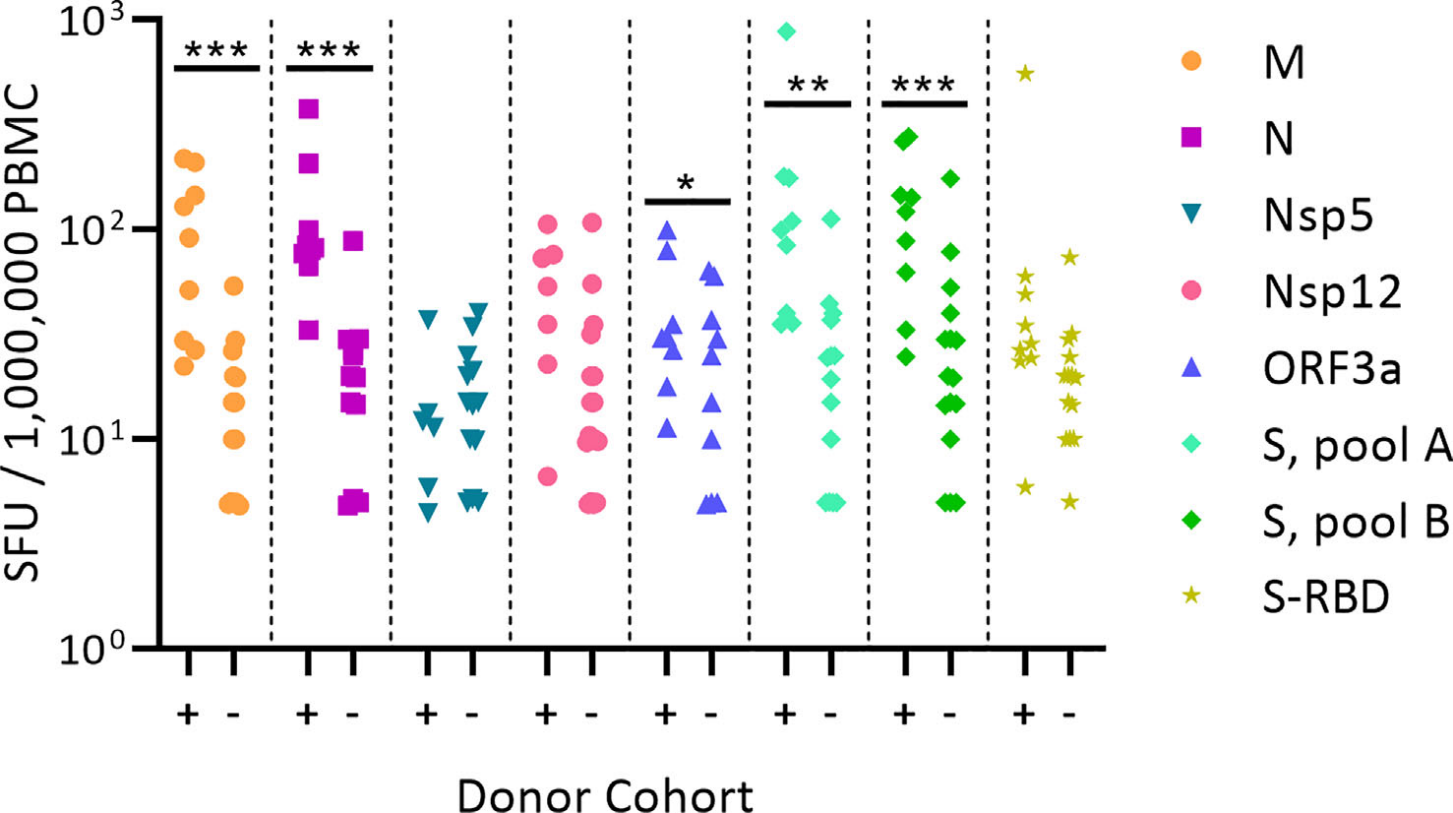
Classic representation of SARS-CoV-2 antigen-specific T cell frequencies in SARS-CoV-2 PCR verified subjects(+) versus Pre-COVID Era individuals(-). PBMC of each individual within the cohort is represented by a dot. The PBMC were challenged with peptide pools covering the SARS-CoV-2 antigens specified on the right. (These peptide pools are closer defined in **Supplementary Table 2**). The individual peptides in each pool were tested at 1.5 µg/ml. An ELISPOT assay was performed measuring the numbers of antigen-induced IFN-y-secreting T cells (spot forming units, SFU) in 200,000 PBMC; following convention, the numbers have been normalized to per million PBMC, as shown on the Y axis. Statistical significance between the two cohorts was determined using an independent samples t test. Significant differences between SARS-CoV-2-infected vs. non-exposed cohorts are marked with * denoting p < 0.05, ** p < 0.01, and *** p < 0.001, respectively.

**Figure 1** shows the test results comparing frequencies of SARS-CoV-2 antigen-reactive IFN-γ-producing T cells in the SARS-CoV-2 PCR-verified (also referred to as COVID-recovered) and Pre-SARS-CoV-2 (hereafter referred to as Pre-COVID) cohorts when tested at a single antigen concentration, 1.5 µg/mL of peptide within each peptide pool. Essentially identical results have been reported by others (9, 12–18) showing that, as a cohort, the frequency of SARS-CoV-2 antigen-specific T cells is significantly elevated in COVID-recovered individuals versus the cohort that has not been infected with the SARS-CoV-2 virus. In all these aforementioned studies, and for T cell immune monitoring in general, it has been of concern however, that such results are inconclusive for the individuals, as a large fraction of COVID-recovered subjects show similar or even lower frequencies of SARS-CoV-2 antigen-specific T cells than the Pre-COVID control subjects. Simple T cell frequency measurements using single SARS-CoV-2 antigens at a single antigen concentration therefore do not permit to reliably distinguish whether an individual has or has not been infected by SARS-CoV-2. This finding, along with the low frequency of SARS-CoV-2 antigen-reactive T cells, may come as a surprise because massive clonal expansions are typically seen initially after infections and vaccinations (61). There is increasing evidence that the SARS-CoV-2 virus actively disrupts the engagement of an immune response (67–73) explaining its weak immunogenicity. The low frequencies of SARS-CoV-2 antigen-reactive T cells in COVID-recovered individuals in turn makes it challenging to unambiguously detect them.

### Accounting for Chance Cross-Reactivity When Testing Suitable Control Mega Peptide Pools to Establish the Background Noise in ELISPOT Assays

The challenge with selecting mega peptide pools that are suited for negative controls (PP. Neg. Contr.) was that we needed to identify antigens to which the test population had not been exposed. As one such antigen we selected the Ebola virus nucleoprotein, and five HIV antigens, as the participating subjects needed to be HIV-seronegative to qualify for this study. In addition, we tested a peptide pool that covered the sequence of the self-antigen, Actin, as a negative control. These candidate PP. Neg. Contr. antigens are listed in **Supplementary Table 2** including the number of 15-mer peptides they contained. Just as for the SARS-CoV-2 peptide pools, all these negative control peptide pool candidates were tested at four concentrations: 1.5 µg/mL, 0.5 µg/mL, 0.17 µg/mL, and 0.06 µg/mL of each peptide within the pool. The number of PP. Neg Contr. candidate-induced IFN-γ-producing cells was compared to the media control, the latter of which was measured in quadruplicate wells. CPI, CERI, and CEFX antigens were measured in singlet, and served as positive controls, respectively (53). For each PBMC sample, the mean and standard deviation (SD) of four replicate media control wells was established and compared to the SFU counts induced by the candidate negative control peptide pools. SFU counts greater than 3 SD of the mean media control counts are highlighted in **Supplementary Table 3**.

The peptide pool covering HIV’s gp160protein induced vigorous SFU formation (> 100 SFU/200,000 PBMC) in 3 subjects’ PBMC, and relatively strong SFU formation (17-42 SFU/200,000 PBMC) in two additional subjects, recalling positive responses at 3-4 consecutive peptide dilutions. At the highest concentration, this peptide pool also elicited elevated SFU numbers in 4 additional subjects. All these subjects who responded to gp160 were HIV-seronegative as established by the blood banks that collected them. The gp160 protein belongs to the p24 superfamily, which shares conserved sequences with related proteins expressed by many retroviruses (74). Thus, cognate cross-reactivity with T cells primed by such retroviruses, rather than chance cross-reactivity, struck us as the likely explanation for the gp160 mega peptide pool-triggered recall responses seen in HIV-seronegative subjects. Be that as it may, the gp160 peptide pool was clearly unsuited as a negative control peptide pool.

The six remaining candidate negative control peptide pools occasionally triggered elevated SFU counts, but these occurred at relatively low frequencies, and predominantly at only the highest peptide concentration (**Supplementary Table 3**). The data therefore provide evidence for low-level chance cross-reactivity when mega peptide pools are tested in ELISPOT assays. In theory, at higher peptide concentrations, this chance cross-reactivity might increase, however, as T cells with low affinity for the peptides might also reach their activation threshold.

Therefore, when analyzing the following SARS-CoV-2 peptide pool-triggered T cell responses, we used and compared two negative controls. One was the conventional “Media control”, established as the mean and SD of 4 replicate wells in which PBMC were cultured with media alone. The second was the PP Neg. Control., calculated as the mean and SD of each donor’s SFU count induced by the six negative control peptide pools at 1.5 µg/mL. Again, these six negative control pools encompassed Ebola, Actin, and the 4 HIV antigen pools (excluding gp160). The mean and SD for the PP Neg. Control, Media control, and the raw data from which these were derived, are specified for each subject in **Supplementary Table 3**.

### Chance Cross-Reactivity Accounts for Most of SARS-CoV-2 Peptide Reactivity in Pre-COVID Era Subjects

We compared the SFU counts induced by SARS-CoV-2 mega peptide pools in the subjects who recovered from mild COVID-19, and those who were bled prior to the COVID era. The SARS-CoV-2 peptide-induced SFU counts were analyzed vs. either the Media control or the PP. Neg. Control in each case, highlighting positive SFU counts as defined by exceeding 3 SD of the respective mean control count, a threshold that identifies positive responses with > 99.6% confidence. As can be seen in **Supplementary Table 4**, in Pre-COVID subjects, the number of SARS-CoV-2 peptide-induced positive SFU counts was significantly lower when control peptide pools were used as to establish the background noise level. Thus, chance cross-reactivity, rather than cognate cross-reactivity with seasonal coronaviruses, accounted for most of the positive responses detected in Pre-COVID control subjects. The few apparently positive cross-reactive responses left after filtering for chance cross-reactivity in this cohort could be discerned from cognate T cell responses to SARS-CoV-2 peptides in SARS-CoV-2 PCR-verified individuals when taking the affinity of the T cell response into account, as will be shown below.

### Affinity for SARS-CoV-2 Peptides Distinguishes Cognate From Cross-Reactive T Cell Recognition

Testing peptide pools in four serial dilutions not only permits generation of confirmatory results without using replicate wells, but also permits one to gain insights into the affinity of the T cells recognizing the respective peptides. In the context of this study, we will distinguish between Level 4 affinity (high affinity, with all four peptide concentrations recalling T cells, color coded in red), Level 3 affinity (intermediate affinity, eliciting a recall response across 3 consecutive peptide dilutions, highlighted in orange), Level 2 affinity (low affinity, only the two highest peptide concentrations elicit a T cell response, color coded in yellow), and Level 1 affinity (borderline low, eliciting a significant T cell response at the highest peptide concentration only, color coded in beige).

As seen in **Table 1**, most COVID-recovered subjects displayed Level 4 (red) affinity T cell responses to several SARS-CoV-2 antigens, while this level was absent in the Pre-COVID Era controls. In the latter, only occasional Level 3 (orange) and Level 2 affinities (yellow) were seen. Thus, high affinity responses to several SARS-Cov-2 antigens (unlike responses detected against individual antigens at a single antigen concentration, see **Figure 1**) appear to be suited to distinguish cognate SARS-CoV-2 specific T cells in COVID-recovered subjects from cross-reactive T cells in subjects infected by other coronaviruses in the Pre-COVID Era. The frequency of SARS-CoV-2 mega peptide pool-specific T cells was low, but clearly elevated > 3 SD over the negative control mega peptide pool control level. As ELISPOT SFU counts follow normal distribution (56), the mean of background plus ≥ 3 SD positivity cut-off definition sets the chances for a single datapoint being a false positive at ≤0.4%; for four responses in a row being false positive, the chances are negligible at a probability of < 0.0256%.

**Table 1:** Affinity analysis of SFU counts triggered by SARS-CoV-2 peptides in PBMC of donors who recovered from PCR-verified SARS- CoV-2 infection **(A)** and in Pre-Covid Era subjects **(B)**.

A									B								
ID.	ORF3a	N	Nsp12	Nsp5	S(A)	S(B)	S-RBD	M	ID.	ORF3a	N	Nsp12	Nsp5	S(A)	S(B)	S-RBD	M
dC1									dP1								
dC2									dP2								
dC3									dP3								
dC4									dP4								
dC5									dP5								
dC6									dP6								
dC7									dP7								
dC8									dP8								
dC9									dP9								
									dP10								
									dP11								
									dP12								
									dP13								
									dP14								
									dP15								
									dP16								
									dP17								
									dP18								

PBMC of 9 subjects with SARS-CoV-2-PCR-verified infection **(A)** and PBMC from 18 subjects from the Pre-Covid Era **(B)** where tested in an ELISPOT assay for reactivity to the specified SARS-CoV-2 mega peptide pools. (These peptide pools are closer defined in**Supplementary Table 2**). All peptide pools were tested in 4 serial dilutions on each PBMC sample at 1.5 ug/mL, 0.5 ug/mL, 0.17 ug/mL, and 0.06ug/mL. Affinity levels are color-coded. Red: high affinity, defined as four consecutive peptide dilutions eliciting a positive recall response with SFU counts exceeding 3 SD of the negative peptide pool-based background. Orange: intermediate affinity, defined as three consecutive peptide dilutions eliciting a positive recall response. Yellow: low affinity, with only the two highest peptide concentrations eliciting positive SFU counts. Beige: only the highest concentration of peptide is positive. The raw counts are provided in **Supplementary Table 5**.

These types of affinity measurements, which rely on serial dilution of peptides and are simple to perform, also require high-throughput suitable test platforms that are frugal with regards to PBMC utilization, such as ELISPOT. To our knowledge, such T cell affinity measurements have so far not been applied systematically to characterize virus-specific T cell responses, thus permitting to compare the above affinity distributions observed for SARS-CoV-2 with other viruses. To compare the SARS-CoV-2 antigen-induced T cell responses with T cell reactivity to a better characterized virus, we also tested mega peptide pools that covered 14 antigens of Epstein Barr Virus (EBV), which commonly infects most humans by the time they have reached adulthood. The raw data are shown in **Supplementary Table 6**. As summarized in **Table 2**, the percentage of EBV mega peptide pools recognized at affinity Levels 1-4 was comparable in both cohorts to the percentage of SARS-CoV-2 peptide pools eliciting Level 1-4 T cell recall responses in the COVID-recovered subjects. SARS-CoV-2 infection, therefore, seems to induce a T cell response that, at least as far as the affinity of nominal antigen-recognition goes, is comparable to the T cell response to EBV.

**Table 2:** Affinity distributions of T cells recognizing SARS-CoV-2- **(A)** vs. EBV peptides **(B)**.

A			SARS-CoV-2 Peptide Pool Positive (%)	
Aff. Level	Definition	Color Code	COVID-Recovered Subjects	Pre-COVID Era Subjects
4	4 serial positives		34%	0%
3	3 serial positives		7%	1%
2	2 serial positives		11%	3%
1	First positive only		11%	3%
B			EBV Peptide Pool Positive (%)	
Aff. Level	Definition	Color Code	COVID-Recovered Subjects	Pre-COVID Era Subjects
4	4 serial positives		31%	17%
3	3 serial positives		7%	6%
2	2 serial positives		7%	6%
1	First positive only		10%	12%

Peptide pools eliciting positive T cell recall responses in the specified affinity level categories are shown as the percentage of all positive responses within the cohort. The raw data are shown in **Supplementary Table 5** for the SARS-CoV-2 peptides, and in **Supplementary Table 6** for the EBV peptides.

### Fine Specificity of SARS-CoV-2 Antigen Recognition in COVID-19-Recovered Subjects

The SARS-CoV-2 peptide pools we used in our study encompassed eight major viral proteins, and systematically covered the respective antigens. Comparing within each donor the SFU counts triggered by these peptide pools permits therefore to assess, first, the total T cell mass mobilized against the virus, and second, which antigens are preferentially targeted by the T cells, i.e., the T cell immune dominance hierarchy within SARS-CoV-2 antigen recognition. As shown in **Table 3**, Spike protein (S) was dominant, or co-dominant, in all COVID-recovered subjects, with 24-51% of all SARS-CoV-2-specific T cells targeting this protein in the individual subjects (it was 40 ± 9% for the cohort). The recognition of Nucleoprotein (N) (18 ± 6%) and the Membrane Protein (M) (16 ± 12%) was next most abundant for the COVID-recovered cohort, while S-RBD (9 ± 6%), Nsp12 (8 ± 6%) and ORF3a (6 ± 4%) peptide pools constituted third tier targets for T cells. There was therefore a clear T cell response hierarchy at the level of the cohort, but it did not always hold up for each individual within the cohort. For subject dC9, for example, only 24% of the SARS-CoV-2-specific T cells targeted S vs. 49% being specific for M, 19% for N, and 8% targeting S-RBD. An immune monitoring effort that focused only on the “immune dominant” S protein would have detected only 24% of the relevant T cells in this subject. In Subject dC2, 40% of the SARS-CoV-2-specific T cells targeted S protein, but these T cells were of lower affinity than the 25% that recognized N. T cell immune monitoring efforts for SARS-CoV-2 therefore ideally should include several, ideally all antigens of the virus, tested in serial dilutions. **Supplementary Table 6** shows for EBV how inaccurate the assessment of T cell immunity to this virus would be if it was restricted to a single antigen, and at a single peptide dose. The same holds for HCMV (75).

**Table 3:** T cell immune dominance of SARS-CoV-2 proteins.

ID.	∑ SFU	ORF3a	N	Nsp12	Nsp5	S (A & B)	S-RBD	M
dC1	191	10%	12%	13%	0%	40%	7%	18%
dC2	267	12%	25%	12%	0%	40%	2%	10%
dC3	230	10%	13%	3%	5%	50%	10%	10%
dC4	264	3%	21%	6%	3%	47%	7%	13%
dC5	216	1%	16%	0%	2%	49%	23%	9%
dC6	332	5%	31%	11%	2%	35%	4%	14%
dC7	253	12%	10%	9%	1%	51%	3%	15%
dC8	184	6%	21%	17%	1%	31%	16%	7%
dC9	81	0%	19%	0%	0%	24%	8%	49%

$\bar{\text{x}}$	na	6%	18%	8%	2%	40%	9%	16%
σ	na	4%	6%	6%	3%	9%	6%	12%
# Pept.	na	66	102	231	74	315	53	53
$\bar{\text{x}}$/(# Pept.)	na	0.09%	0.18%	0.03%	0.03%	0.13%	0.17%	0.30%

The total SARS-CoV-2-specific T cell mass (∑SFU) was calculated by adding up for each SARS-CoV-2-recovered donor the numbers of SFU elicited by all SARS-CoV-2 peptide pools in that donor at 1.5 µg/mL (see the raw data in **Supplementary Table 5**). In the top panel, the percentage of T cells targeting each of the SARS-CoV-2 antigens is shown relative to the total clonal SARS-CoV-2-specific T cell mass in that individual, representing an immune dominance index. The superimposed heatmap specifies the affinity level of the respective T cell population, with the color code defined in **Table 2**. The lower panel shows the mean percentage ($\bar{\text{x}}$) *and SD of this immunodominance index for the cohort. Addressing the hypothesis that T cell immune dominance of a SARS-CoV-2 antigen is related to its size, the number of peptides in each pool is shown (# Pept.) and the mean immune dominance index (* $\overset{¯}{x}$ *) is normalized for the number of peptides (* $\bar{\text{x}}$ */(# Pept).*

One possible explanation for the relative immune dominance of S protein over the other SARS-CoV-2 proteins is its size relative to the others. The longer a protein, the more potential T cell epitopes it contains. S protein was covered by 315 peptides vs. for example N, one third as long, which was covered by 102 peptides, and M, half as long as N, with 53 peptides. Indeed, for these three antigens, and also for S-RBD and ORF3a, the percentage of T cells targeting them divided by the number of peptides present in each pool (corresponding to the length of the respective protein) gave numbers in the same ballpark: 0.13%, 0.18%, 0.3%, 0.17%, and 0.09% for S, N, M, S-RBD, and ORF3a, respectively (**Table 3**). For these SARS-CoV-2 antigens, therefore, the magnitude of T cell response targeting each appeared to be a mere function of the proteins’ respective sizes. With this ratio substantially lower, at 0.03%, Nsp12and Nsp5 were under-targeted relative to their size, possibly suggesting that the expression levels of these two antigens is lower during SARS-CoV-2 replication than that of the other SARS-CoV-2 antigens.

### Non-Cross-Reactive T Cell Recognition of Seasonal Coronavirus Spike Proteins

People around the world commonly get infected with seasonal coronaviruses causing common cold (CCC) such as 229E, NL63, OC43, and HKU1, and over the years most adults can be expected to have been infected with several of these CCC strains. T cell reactivity induced by SARS-CoV-2 mega peptide pools in individuals who clearly have not been exposed to SARS-CoV-2 have therefore been attributed to cognate cross-reactivity with CCC. By introducing negative control mega peptide pools to account for noise created by chance cross-reactivity (**Supplementary Table 3**), and by adding the requirement for high affinity T cell recognition (**Table 1**), we show that SARS-CoV-2 antigens are not recognized by subjects in the Pre-COVID cohort as a consequence of cross-reactivity with CCC antigens. We therefore asked the reverse question: do mega peptide pools that specifically cover CCC antigens detect T cell memory in both cohorts?

We tested Spike proteins of 229E, NL63, OC43, and HKU1, which, due to their size, were each represented in two mega peptide pools (as was the SARS-CoV-2 Sprotein itself). These CCC peptide pools were also tested at four concentrations, following exactly the same protocol as specified above for the SARS-CoV-2 S protein, and all other peptide pools tested in this study. While no Level 4 affinity response to SARS-CoV-2 S protein peptides were seen in Pre-COVID-19 subjects (**Table 1B**), eight of eighteen subjects in this cohort (44%) displayed high affinity T cell responses to at least one of these CCC peptide pools with seven of eighteen (39%) showing no response at all (**Table 4B**). In the PCR-verified cohort seven of nine subjects (78%) showed high affinity responses for CCC peptide pools and none displayed no response at all (**Table 4A**). In isolation, these data could be interpreted as evidence for T cells primed by SARS-CoV-2 infection being cross-reactively recalled by CCC S antigens. However, Pre-COVID-19 donors do not show SARS-CoV-2 S-antigen reactivity (**Table 1B**) in spite of their reactivity to CCC S antigens (**Table 4B**), and the frequency of CCC S antigen-specific T cells is not elevated in the recently SARS-CoV-2-infected cohort (SFU counts in **Table 4A vs. B**) as would be expected in the case of a cross-reactive boost. Therefore, we conclude that cross-reactivity does appear to play a major role in shaping the respective S-antigen-specific T cell repertoires.

**Table 4:** T cell recall responses to Spike proteins of the four Common Cold Coronaviruses, 229E, NL63, OC43, and HKU1, each represented due to size in two peptide pools.

A									B								
ID.	HKU1 S (A)	HKU1 S (B)	229E S (A)	229E S (B)	NL63 S (A)	NL63 S (B)	OC43 S (A)	OC43 S (B)	ID.	HKU1 S (A)	HKU1 S (B)	229E S (A)	229E S (B)	NL63 S (A)	NL63 S (B)	OC43 S (A)	OC43 S (B)
dC1									dP1								
dC2									dP2								
dC3									dP3								
dC4									dP4								
dC5									dP5								
dC6									dP6								
dC7									dP7								
dC8									dP8								
dC9									dP9								
									dP10								
									dP11								
									dP12								
									dP13								
									dP14								
									dP15								
									dP16								
									dP17								
									dP18								

Each peptide pools has been tested in the specified four concentrations in a standard IFN-γ ELISPOT assay. SFU counts exceeding 3 SD of the mean of the negative peptide pool control are highlighted according to T cell affinity levels, as specified in **Table 2**. The original counts can be found in **Supplementary Table 7**.

Our data showing the specificity of T cell responses to SARS-CoV-2 and CCC in the absence of major cross-reactivities are in line with other reports that also relied on IFN-γ ELISPOT assays for T cell detection (14, 19, 20) and contradict publications that claim high cross-reactivity using general T cell activation measurements. It therefore seems possible that the method of observation itself might affect the results. While these T cell assays have, to our knowledge, not been thoroughly compared so far, a recent study might shed light on this discrepancy (19). This group relied on different T cell assays to study SARS-CoV-2-infected subjects vs. individuals without known exposure to the virus. Using IFN-γ ELISPOT, SARS-CoV-2 antigen-triggered responses were found to be specific, commonly occurring in those who had been infected but rarely in unexposed subjects. By contrast, over 90% of individuals in both groups showed proliferation and cellular lactate responses to S subunits S1/S2. The authors concluded that the detection of T cell responses to SARS-CoV-2 is therefore critically dependent on the choice of assay and antigen. The ELISPOT assay used detects IFN-γ-producing effector memory cells that can directly engage in defense reactions, but it does not detect the precursor cells for effector memory cells such as naïve and stem cell-like memory cells, which do not produce effector cytokines (61, 76). Assays that measure T cell activation in general do not distinguish between such T cell subpopulations.

## Concluding Remarks

The overall question that we addressed was whether test conditions can be established that permit to clearly identify SARS-CoV-2-specific T cell memory engaged in individuals who underwent a mild infection vs. humans who have not been infected with this virus. Previous publications on this subject matter reported up to 80% false positive results for uninfected individuals due to alleged T cell cross-reactivity. Here we have established criteria by which false positive results can be reduced to 0% (0 of 18 Pre-COVID Era test subjects), while permitting the detection of SARS-CoV-2-reactive T cells in eight of nine (89%) SARS-CoV-2 PCR-verified subjects. To accomplish this discrimination, a combination of four criteria needed to be used. First, the detection of *ex vivo* IFN-γ-producing effector memory T cells was required. Second, we introduced negative control mega peptide pools, instead of media Suppl alone, to establish the background noise level caused by chance cross-reactivity. Third, we introduced the criterion that a T cell response scores positive only if it has sufficient affinity, being triggered by at least four 1 + 2 (vol + vol) (3-fold) serial dilutions of the test peptides. Lastly, as COVID-recovered donors responded to several SARS-CoV-2 antigens, a broad, multi-antigen-specific T cell response profile was established as a requirement for scoring a subject positive. In addition to exhibiting a high affinity T cell response to at least one mega peptide pool, a second high or intermediate affinity level T cell response was also identified in 89% our SARS-CoV-2 PCR-verified cohort. To meet the latter requirement of multi-specificity, several SARS-CoV-2 antigens need to be tested, as there is no fixed immune dominance pattern among them, reminiscent of the T cell response to EBV (**Supplementary Table 6**) and HCMV (75).

Following infection with the original SARS coronavirus (SARS-CoV), antibody and B cell memory wanes, but evidence for T cell memory remains (77). Antibody titers, and potentially B cell memory, also appear to be short-lived after SARS-CoV-2 infection (78), but it is presently not known whether T cell memory to this virus will be durably maintained. If serum antibody reactivity fails to provide reliable information on previous exposure to SARS-CoV-2, then potentially T cell diagnostics could fill this gap.

Even though we report here that SARS-CoV-2- and EBV-specific T cells occur in similar frequencies in COVID-recovered subjects (See **Table 1** vs. **Supplementary Table 6**), it might be premature to conclude that such findings signify the induction of a robust cognate T cell response following SARS-CoV-2 infection. Cognate T cell responses in general show a typical kinetic: in the first weeks after the onset of infection, the frequency of the antigen-specific T cells reaches a peak, after which the frequencies drop to a substantially lower steady state level (78). We measured frequencies of SARS-CoV-2 antigen-specific T cells near their expected peak in our COVID-recovered cohort, while the frequencies of the EBV-specific T cells were assessed in steady state. Therefore, in light of the typical T cell response kinetic, the data reported here, and supported by existing literature, may also signify that mild/asymptomatic SARS-CoV-2 infection induces a much weaker T cell response than natural EBV infection, or other viruses against which we develop protective immunity. The already low numbers of SARS-CoV-2-specific T cells early on after a mild/asymptomatic infection might further decrease with time, which needs to be established. Being able to accurately detect such rare SARS-CoV-2-specific T cells is an important step for immune diagnostics, but is just the first step toward understanding their role in host defense.

## Data Availability Statement

The datasets generated in this study will be made available by the authors, without undue reservation, to any qualified researcher.

## Ethics Statement

The studies involving human participants were reviewed and approved by Advarra IRB Approved # Pro00043178, CTL study number: GL20-16 entitled COVID 19 Immune Response Evaluation. The patients/participants provided their written informed consent to participate in this study.

## Author Contributions

Experiments were designed by AAL, PL, and PR. Experimental data were generated by AAL, TZ, and GK. This publication serves as part of AAL’s doctoral thesis to be submitted to the Universidad Complutense de Madrid, Madrid, Spain. All authors contributed to the article and approved the submitted version.

## Funding

This study was funded by the R&D budget of Cellular Technology Limited.

## Conflict of Interest

PL is Founder, President and CEO of Cellular Technology Ltd., a company that specializes in immune monitoring by ELISPOT testing, producing high-throughput-suitable readers, test kits, and GLP-compliant contract research. AAL, GK, and TZ are employees of CTL. This study was funded by CTL, and the funder directed the study design, collection, analysis, interpretation of data, the writing of this article, and made the decision to submit it for publication.
