# Supplementary material for: Deconvoluting the T Cell Response to SARS-CoV-2: Specificity Versus Chance and Cognate Cross-Reactivity
Source: Front Immunol. 2021 May 28;12:635942. doi: 10.3389/fimmu.2021.635942 (PMC8196231; doi:10.3389/fimmu.2021.635942)
Supplement: Supplementary file 1 [file DataSheet_1.zip › PDF's of All S Material/S Figure 1.pdf]

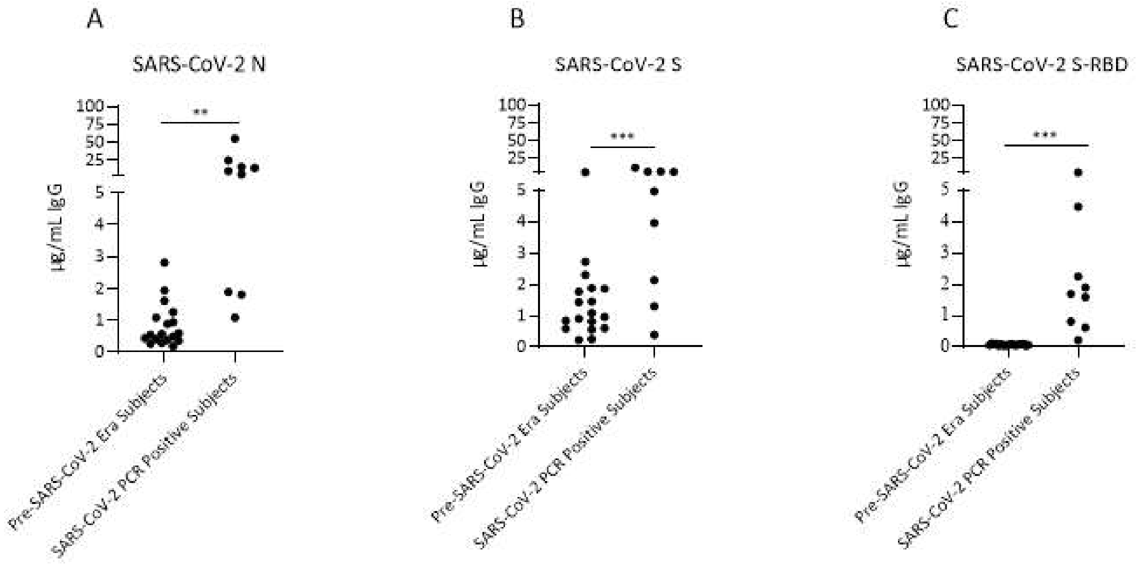

S. FIGURE 1. IgG antibody reactivity against SARS-CoV-2 antigens. Plasma from 18 Pre-SARS-CoV-2 Era Subjects and 9 individuals who underwent mild PCR-confirmed SARS-CoV-2 infection (SARS-CoV-2 PCR- Positive Subjects) were assessed for IgG antibody against A) SARS-CoV-2 N protein, B) the truncated SARS-CoV-2 S fragment, S1, or C) the S-RBD fragment. ELISA binding signal was then interpolated into µg/mL IgG equivalents using a reference standard. Each serum sample is represented by a dot. Statistical significance between the two cohorts was determined using an unpaired Student's t-test. Significant differences between cohorts are marked with ** denoting $p < 0.01$, and *** $p < 0.001$, respectively.
