# Supplementary material for: Deconvoluting the T Cell Response to SARS-CoV-2: Specificity Versus Chance and Cognate Cross-Reactivity
Source: Front Immunol. 2021 May 28;12:635942. doi: 10.3389/fimmu.2021.635942 (PMC8196231; doi:10.3389/fimmu.2021.635942)
Supplement: Supplementary file 1 [file DataSheet_1.zip › PDF's of All S Material/S Figure 2.pdf]

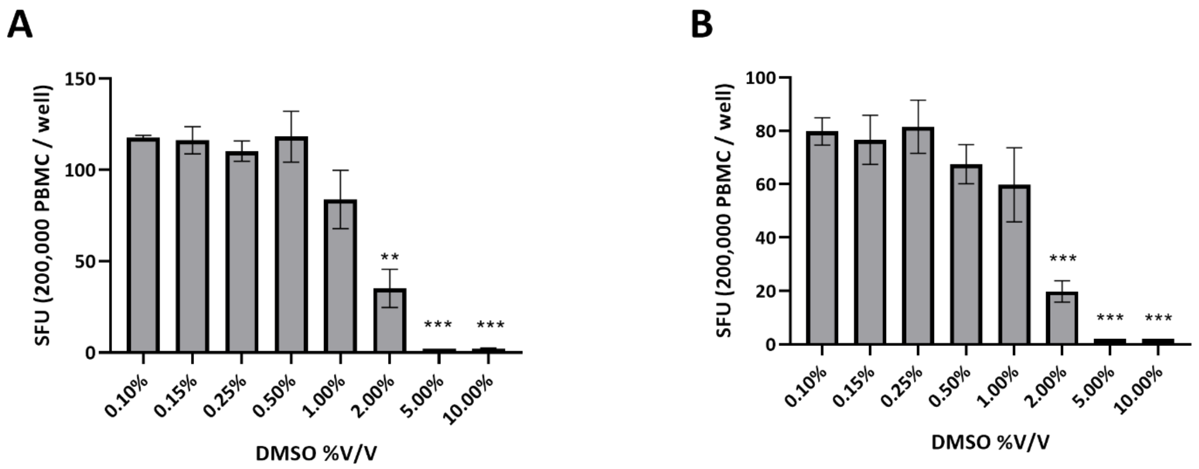


**S. FIGURE 2.** The influence of dimethyl sulfoxide (DMSO) on the T cell recall response as measured in a standard IFN-γ ELISPOT assay. DMSO is a solvent commonly used for the resuspension of mega peptide pools. It was present at 0.24% (vol/vol) in all T cell activation cultures when mega peptide pools were tested for this publication. In this figure we address DMSO's potential interference with the test results by performing the assay in the presence of increasing concentrations of DMSO, as specified on the X axis. PBMC of subject dp10 **(A)** and dp14 **(B)** were tested for reactivity to EBV-MHC Class I Control Peptide Pool (available from C.T.L Catalog #PA-EBV-001). Except for the DMSO concentration, all other assay conditions were kept constant and matched those applied throughout this publication: the PBMC were at 200,000 PBMC per well and were cultured for 24h in the presence of 1.5 µg/ml peptides before the cell culture was terminated and the IFN-γ SFU visualized. Each bar shows the mean SFU counts and the standard deviation for three replicate wells. Taking the 0.1% DMSO concentration as the reference value, the statistical significance of inhibition was determined using Dunnett's multiple comparisons test. Significant differences are marked with ** denoting $p < 0.01$, and *** $p < 0.001$, respectively.
