# Supplementary material for: Deconvoluting the T Cell Response to SARS-CoV-2: Specificity Versus Chance and Cognate Cross-Reactivity
Source: Front Immunol. 2021 May 28;12:635942. doi: 10.3389/fimmu.2021.635942 (PMC8196231; doi:10.3389/fimmu.2021.635942)
Supplement: Supplementary file 1 [file DataSheet_1.zip › PDF's of All S Material/S Table 1.pdf]

A

Demographic Characteristics								Response to Pos. Ctrl.		
ID	PCR Confirmed	Hospitalization Yes or No	Days from verification to collection	Date of Collection	Race	Gender	Age	CPI (SFU)	CERI (SFU)	CEFX (SFU)
dC1	Pos	No	14	05/11/20	African/American	Male	49	201	18	36
dC2	Pos	No	14	05/11/20	African/American	Female	20	>500	90	92
dC3	Pos	No	34	06/11/20	Caucasian	Female	28	391	371	186
dC4	Pos	No	34	7/7/2020	Caucasian	Female	24	925	121	68
dC5	Pos	No	24	7/20/2020	African/American	Male	33	143	170	69
dC6	Pos	No	20	7/22/2020	Hispanic/Latino	Female	25	>500	56	63
dC7	Pos	No	17	05/14/20	African/American	Female	22	>500	272	70
dC8	Pos	Yes	31	7/6/2020	Caucasian	Female	53	404	160	396
dC9	Pos	No	95	07/08/20	Caucasian	Female	51	87	45	43

B

Demographic Characteristics					Response to Pos. Ctrl.		
ID	Date of Collection	Race	Gender	Age	CPI (SFU)	CERI (SFU)	CEFX (SFU)
dP1	3/15/2017	Hispanic	Male	18	181	60	95
dP2	9/27/2017	rican/Americ	Female	28	453	120	153.5
dP3	7/26/2017	Hispanic	Male	34	68	108	120
dP4	5/16/2018	Hispanic	Male	37	163	>500	456
dP5	10/16/2018	Hispanic	Male	30	193	202	188
dP6	10/1/2018	Caucasian	Female	34	19	11	18
dP7	3/14/2018	Hispanic	Male	31	394	194	192
dP8	8/16/2017	Hispanic	Female	39	279	180	146
dP9	8/13/2018	rican/Americ	Male	49	65	32	93
dP10	7/20/2016	Caucasian	Female	43	>500	265	303
dP11	8/22/2019	Caucasian	Female	22	427	360	>500
dP12	7/15/2019	Hispanic	Male	24	255	>500	>500
dP13	6/12/2019	Hispanic	Female	52	>500	272	221
dP14	5/6/2019	ispanic/Latin	Female	46	>500	159	188
dP15	4/10/2019	ispanic/Latin	Male	36	267	358	369
dP16	11/2/2011	Hispanic	Male	42	>500	321	274
dP17	7/8/2019	Caucasian	Male	54	438	66	77
dP18	7/16/2017	Hispanic	Male	45	134	81	142

S. TABLE 1. Human subjects tested in this study and their PBMC's response to positive control antigens. The SARS-CoV-2-PCR-verified cohort **(A)** consisted of nine individuals who underwent mild PCR-confirmed SARS-CoV-2 infection (subject IDs dC1-9, for donors recovered from COVID-19). The Pre-COVID Era cohort **(B)** consisted of 18 subjects who were bled prior to December 2019 (subject IDs dP1-18, for donor Pre-COVID-19 Era). The PBMC of these subjects were tested in a standard IFN-γ ELISPOT assay for reactivity to the positive control antigens CPI, CERI and CEFX as specified in Materials and Methods. The SFU counts induced in 200,000 PBMC are shown whereby >500 denotes that SFUs were too numerous to count due to confluence.
