# Supplementary material for: Deconvoluting the T Cell Response to SARS-CoV-2: Specificity Versus Chance and Cognate Cross-Reactivity
Source: Front Immunol. 2021 May 28;12:635942. doi: 10.3389/fimmu.2021.635942 (PMC8196231; doi:10.3389/fimmu.2021.635942)
Supplement: Supplementary file 1 [file DataSheet_1.zip › PDF's of All S Material/S Table 2.pdf]

Peptide Pools for SARS-CoV-2 Proteins				
Protein name	Origin	Protein-ID	# of Peptides	Product Code
ORF3a	SAR-CoV-2	P0DTC3	66	PM-WCPV-AP3A
Nuceloprotein (N)		P0DTC9	102	PM-WCPV-NCAP
Nsp12		P0DTC9:4393-5324	231	PM-WCPV-Nsp12-1
Nsp5		P0DTC1:3264-3569	74	PM-WCPV-Nsp5-1
Spike (S) A		P0DTC2	158	PM-WCPV-S
Spike (S) B			157	
S-RBD		P0DTC2:319-541	53	PM-WCPV-S-RBD-1
Membrane (M)		P0DTC5	53	PM-WCPV-VME

Peptide Pools for EBV Proteins				
Protein name	Origin	Protein-ID	# of Peptides	Product Code
BARF1	EBV	P03228	53	PM-C-EBV-2
BMLF1		Q04360	117	
BMRF1		P03191	99	
BRLF1		P03209	149	
BZLF1		P03206	59	
EBNA-LP		Q8AZK7	124	
EBNA1		P03211	158	
EBNA2		P12978	19	
EBNA3a		P12977	234	
EBNA3b		Q1HVG4	279	
EBNA3c		Q69140	265	
GP350/340		P03200	224	
LMP1		PO3230	94	
LMP2		P13285	122	

Candidate Negative Control Peptide Pools				
Protein name	Origin	Protein-ID	# of Peptides	Product Code
Nef Protein	HIV	LAN*	150	PM-HIV-NEF
Gag polyprotein		LAN*	150	PM-HIV-GAG
Pol Polyprotein		LAN*	150	PM-HIV-POL
Con B gag motif		LAN*	123	PM-HIV-CONB
gp160		LAN*	150	PM-HIV-ENV
Actin	Human	P68133**	92	PM-ACTS
Nucleoprotein (N)	Ebola-Thai	B8XCN6**	182	PM-TEBOV-NP

Peptide Pools for S Proteins of Common Cold Coronaviruses (CCC)				
Origin	Protein-ID	Product Code	Peptide Pool	# of Peptides
HKU1	Q5MQD0	PM-HKU1-S-1	HKU-1 S(A)	169
			HKU-1 S(B)	168
HCoV-229E	P15423	PM-229E-S-1	229E S(A)	146
			229E S(B)	145
HCoV-NL63	Q6Q1S2	PM-NL63-S-1	NL63 S(A)	169
			NL63 S(B)	168
HCoV-OC43	P36334	PM-OC43-S-1	OC43 S(A)	168
			OC43 S(B)	168

S. TABLE 2. Definition of mega peptide pools used in this study. All peptide pools consisted of unpurified 15-mer peptides that systematically cover the entire amino acid (a.a) sequence of the respective protein in steps of 4 a.a. with 11 a.a. overlaps. The number of peptides in each mega peptide pool is specified. All of these peptide pools were purchased and are commercially available from JPT.
