# Supplementary material for: Deconvoluting the T Cell Response to SARS-CoV-2: Specificity Versus Chance and Cognate Cross-Reactivity
Source: Front Immunol. 2021 May 28;12:635942. doi: 10.3389/fimmu.2021.635942 (PMC8196231; doi:10.3389/fimmu.2021.635942)
Supplement: Supplementary file 1 [file DataSheet_1.zip › PDF's of All S Material/S Table 3.pdf]

A

ID.	[ ]	HIV - NEF	HIV - Gag	HIV - Pol	HIV - Con B Gag	HIV - gp160	Human - Actin	Ebola - Thai N	Media Controls: Media	$\bar{x}$	σ	$\bar{x}$+3σ	PP Neg. Ctrl.: $\bar{x}$	σ	$\bar{x}$+3σ
dC1	1.5 ug/mL	0	0	0	3	1	1	0	8	2.00	4.00	14.00	0.67	1.21	4.30
	0.5 ug/mL	0	0	0	2	0	2	0	0						
	0.17ug/mL	0	1	0	0	1	1	0	0						
	0.06 ug/mL	0	0	0	0	0	0	0	0						
dC2	1.5 ug/mL	0	0	3	0	2	0	3	1	1.00	0.82	3.45	1.00	1.55	5.65
	0.5 ug/mL	2	2	1	1	1	0	1	0						
	0.17ug/mL	**5**	0	0	0	1	0	2	2						
	0.06 ug/mL	0	1	2	1	2	1	0	1						
dC3	1.5 ug/mL	**3**	1	**2**	0	**9**	0	1	0	0.25	0.50	1.75	1.17	1.17	4.67
	0.5 ug/mL	1	1	0	0	**5**	0	**4**	1						
	0.17ug/mL	0	0	0	**2**	1	0	1	0						
	0.06 ug/mL	1	**2**	1	0	**2**	1	1	0						
dC4	1.5 ug/mL	1	2	2	1	0	1	0	2	0.75	0.96	3.62	1.17	0.75	3.42
	0.5 ug/mL	2	0	3	2	0	1	1	1						
	0.17ug/mL	**5**	**6**	3	3	**4**	2	3	0						
	0.06 ug/mL	3	3	3	1	1	1	2	0						
dC5	1.5 ug/mL	**8**	6	0	2	**27**	5	1	4	1.50	1.91	7.24	3.67	3.14	13.09
	0.5 ug/mL	**10**	7	3	4	**23**	5	3	0						
	0.17ug/mL	6	2	2	7	**8**	5	7	0						
	0.06 ug/mL	2	3	1	0	3	2	5	2						
dC6	1.5 ug/mL	3	1	4	1	**17**	3	3	0	1.75	1.26	5.52	2.50	1.22	6.17
	0.5 ug/mL	1	1	**34**	0	**7**	2	5	3						
	0.17ug/mL	2	1	2	2	**9**	**9**	**6**	2						
	0.06 ug/mL	**8**	**6**	3	3	**9**	3	4	2						
dC7	1.5 ug/mL	2	2	**16**	0	**8**	1	2	0	1.25	1.26	5.02	3.83	6.01	21.87
	0.5 ug/mL	0	1	1	2	5	1	3	1						
	0.17ug/mL	0	0	0	2	1	**9**	1	1						
	0.06 ug/mL	0	4	1	2	4	3	0	3						
dC8	1.5 ug/mL	0	2	0	1	1	1	0	0	1.00	1.41	5.24	0.67	0.82	3.12
	0.5 ug/mL	1	1	0	1	0	1	2	1						
	0.17ug/mL	1	0	3	3	1	2	0	3						
	0.06 ug/mL	0	3	4	1	0	5	2	0						
dC9	1.5 ug/mL	0	0	0	**1**	0	0	**1**	0	0.00	0.00	0.00	0.33	0.52	1.88
	0.5 ug/mL	0	0	0	**3**	0	0	**1**	0						
	0.17ug/mL	0	0	**1**	0	0	0	0	0						
	0.06 ug/mL	0	0	0	0	0	**2**	0	0						

B

ID.	[ ]	HIV - NEF	HIV - Gag	HIV - Pol	HIV - Con B Gag	HIV - gp160	Human - Actin	Ebola - Thai N	Media Controls: Media	$\bar{x}$	σ	$\bar{x}$+3σ	PP Neg. Ctrl.: $\bar{x}$	σ	$\bar{x}$+3σ
dP1	1.5 ug/mL	**4**	2	2	**4**	**3**	1	**4**	1	1.25	0.50	2.75	2.83	1.33	6.82
	0.5 ug/mL	**3**	**4**	**5**	0	**5**	2	1	2						
	0.17ug/mL	2	2	**5**	0	**3**	**3**	**4**	1						
	0.06 ug/mL	**6**	1	1	2	2	2	**6**	1						
dP2	1.5 ug/mL	2	3	1	0	2	2	3	0	0.50	1.00	3.50	1.83	1.17	5.34
	0.5 ug/mL	3	**5**	2	1	3	0	**4**	0						
	0.17ug/mL	2	2	1	1	0	0	3	2						
	0.06 ug/mL	0	3	**4**	2	0	2	0	0						
dP3	1.5 ug/mL	0	0	1	0	0	0	2	0	1.50	1.73	6.70	0.50	0.84	3.01
	0.5 ug/mL	1	2	1	2	4	3	1	3						
	0.17ug/mL	0	4	4	4	2	1	3	3						
	0.06 ug/mL	0	1	0	0	5	2	3	0						
dP4	1.5 ug/mL	0	3	0	6	8	0	4	0	2.50	2.89	11.16	2.17	2.56	9.85
	0.5 ug/mL	3	2	1	6	5	4	6	5						
	0.17ug/mL	1	3	1	4	5	2	2	5						
	0.06 ug/mL	1	3	2	3	1	3	6	0						
dP5	1.5 ug/mL	**11**	4	1	2	0	5	3	3	1.25	1.26	5.02	4.33	3.56	15.01
	0.5 ug/mL	1	2	1	3	4	2	2	1						
	0.17ug/mL	5	4	1	4	2	3	4	0						
	0.06 ug/mL	1	0	2	1	1	1	0	1						
dP6	1.5 ug/mL	4	5	**10**	**7**	6	**7**	6	4	1.75	1.71	6.87	6.50	2.07	12.72
	0.5 ug/mL	**7**	6	**7**	6	3	**8**	5	1						
	0.17ug/mL	4	4	3	4	4	5	3	2						
	0.06 ug/mL	3	5	4	3	2	4	**9**	0						
dP7	1.5 ug/mL	**4**	1	1	2	**20**	2	0	1	0.75	0.50	2.25	1.67	1.37	5.77
	0.5 ug/mL	1	1	1	1	**10**	1	1	1						
	0.17ug/mL	0	1	1	2	**3**	2	**4**	0						
	0.06 ug/mL	1	1	2	**3**	2	2	1	1						
dP8	1.5 ug/mL	1	4	3	2	2	3	2	1	2.50	3.11	11.83	2.50	1.05	5.65
	0.5 ug/mL	0	3	0	1	9	1	3	2						
	0.17ug/mL	2	3	5	2	6	3	11	0						
	0.06 ug/mL	0	3	3	5	3	2	7	7						
dP9	1.5 ug/mL	**5**	2	3	1	**20**	1	**8**	2	0.50	1.00	3.50	3.33	2.73	11.53
	0.5 ug/mL	0	2	0	1	3	1	1	0						
	0.17ug/mL	2	2	3	2	0	0	1	0						
	0.06 ug/mL	2	2	0	2	0	0	1	0						
dP10	1.5 ug/mL	**25**	0	0	0	**146**	1	1	0	0.25	0.50	1.75	4.50	10.05	34.66
	0.5 ug/mL	**6**	0	0	0	**89**	0	0	1						
	0.17ug/mL	**2**	0	0	0	**57**	0	1	0						
	0.06 ug/mL	1	0	1	0	**12**	0	1	0						
dP11	1.5 ug/mL	1	**5**	1	0	2	0	2	1	0.50	0.58	2.23	1.50	1.87	7.11
	0.5 ug/mL	1	**4**	0	1	1	1	**3**	0						
	0.17ug/mL	0	1	0	0	1	0	2	0						
	0.06 ug/mL	0	**4**	1	1	1	1	0	1						
dP12	1.5 ug/mL	**20**	1	0	2	**148**	1	0	1	1.00	0.82	3.45	4.00	7.87	27.62
	0.5 ug/mL	**10**	0	1	0	**87**	0	0	1						
	0.17ug/mL	1	2	0	1	**27**	0	1	0						
	0.06 ug/mL	2	0	0	1	**11**	0	1	2						
dP13	1.5 ug/mL	4	0	4	0	1	1	3	0	1.00	1.41	5.24	2.00	1.90	7.69
	0.5 ug/mL	2	1	2	3	2	1	3	0						
	0.17ug/mL	0	0	2	1	2	3	1	1						
	0.06 ug/mL	0	2	0	2	0	2	5	3						
dP14	1.5 ug/mL	9	6	7	6	7	6	10	2	4.25	3.30	14.16	7.33	1.75	12.59
	0.5 ug/mL	3	6	1	3	4	4	7	8						
	0.17ug/mL	1	3	2	6	7	2	3	1						
	0.06 ug/mL	4	7	3	3	1	5	5	6						
dP15	1.5 ug/mL	6	4	5	6	7	2	6	5	4.75	2.87	13.37	4.83	1.60	9.64
	0.5 ug/mL	2	4	2	3	1	1	4	1						
	0.17ug/mL	5	4	3	4	2	3	3	5						
	0.06 ug/mL	9	5	1	2	1	2	3	8						
dP16	1.5 ug/mL	11	8	17	7	17	18	11	18	15.67	4.04	27.79	12.00	4.56	25.68
	0.5 ug/mL	4	4	7	6	15	6	9	18						
	0.17ug/mL	5	4	3	8	16	3	5	11						
	0.06 ug/mL	7	4	7	7	18	8	6	56						
dP17	1.5 ug/mL	2	4	2	4	1	1	2	0	3.50	3.87	15.12	2.50	1.22	6.17
	0.5 ug/mL	2	1	2	3	1	2	5	3						
	0.17ug/mL	2	0	3	0	1	1	0	2						
	0.06 ug/mL	4	1	0	4	4	3	2	9						
dP18	1.5 ug/mL	4	**14**	3	**15**	**8**	**12**	**10**	2	2.25	1.71	7.37	9.67	5.09	24.92
	0.5 ug/mL	**12**	**11**	3	4	6	**14**	**9**	4						
	0.17ug/mL	1	5	7	7	**8**	7	**11**	0						
	0.06 ug/mL	6	3	6	3	2	3	2	3						

S. Table 3. IFN-γ SFU counts triggered by candidate negative control mega peptide pools. PBMC of 9 subjects with SARS-CoV-2-PCR-verified infection **(A)** and PBMC from 18 subjects from the Pre-Covid Era **(B)** where tested in an ELISPOT assay for reactivity to the specified candidate negative control mega peptide pools. (These peptide pools are closer defined in S. Table 2.) All peptide pools were tested in 4 serial dilutions on each PBMC sample in the concentrations specified under [ ]. Highlighted are SFU counts that exceeded the mean of the four media control SFU counts by more than 3 SD for the respective subject, as listed under “Media Control”. The mean, SD and 3 SD for the Peptide Negative Control (PP Neg. Cont.) is also shown for each subject as calculated from the SFU counts triggered by the six negative control peptide pools at 1.5 µg/mL excluding gp160 for reasons specified in the Text.
