# Supplementary material for: Deconvoluting the T Cell Response to SARS-CoV-2: Specificity Versus Chance and Cognate Cross-Reactivity
Source: Front Immunol. 2021 May 28;12:635942. doi: 10.3389/fimmu.2021.635942 (PMC8196231; doi:10.3389/fimmu.2021.635942)
Supplement: Supplementary file 1 [file DataSheet_1.zip › PDF's of All S Material/S Table 4.pdf]

A

ID.	[ ]	ORF3a	N	Nsp12	Nsp5	S (A)	S (B)	S-RBD	M	Media Controls: Media	$\bar{x}$	σ	$\bar{x}$+3σ
dP1	1.5 ug/mL	0	5	2	3	5	8	4	2	1	1.25	0.50	2.75
	0.5 ug/mL	0	3	3	1	6	7	8	1	2			
	0.17ug/mL	0	3	4	0	3	7	0	2	1			
	0.06 ug/mL	1	5	3	6	2	5	5	1	1			
dP2	1.5 ug/mL	0	0	3	3	1	6	2	1	0	0.50	1.00	3.50
	0.5 ug/mL	2	2	1	2	3	0	4	1	0			
	0.17ug/mL	4	2	2	1	2	3	2	3	2			
	0.06 ug/mL	2	3	2	1	0	2	1	3	0			
dP3	1.5 ug/mL	0	4	7	1	1	6	3	1	0	1.50	1.73	6.70
	0.5 ug/mL	2	2	10	0	4	4	1	3	3			
	0.17ug/mL	1	1	4	1	5	2	1	1	3			
	0.06 ug/mL	4	1	3	0	1	3	1	1	0			
dP4	1.5 ug/mL	12	6	11	5	8	35	6	3	0	2.50	2.89	11.16
	0.5 ug/mL	15	14	11	7	7	28	0	13	5			
	0.17ug/mL	4	7	6	10	4	7	1	8	5			
	0.06 ug/mL	6	12	5	1	4	4	2	1	0			
dP5	1.5 ug/mL	5	1	3	2	1	1	0	0	3	1.25	1.26	5.02
	0.5 ug/mL	2	0	0	2	1	3	1	4	1			
	0.17ug/mL	1	1	2	3	0	1	1	3	0			
	0.06 ug/mL	2	2	2	1	2	0	2	2	1			
dP6	1.5 ug/mL	2	3	4	4	3	1	4	1	4	1.75	1.71	6.87
	0.5 ug/mL	2	4	5	6	3	4	8	2	1			
	0.17ug/mL	4	5	2	3	0	4	1	6	2			
	0.06 ug/mL	0	3	2	6	6	3	3	2	0			
dP7	1.5 ug/mL	3	0	4	1	2	2	4	3	1	0.75	0.50	2.25
	0.5 ug/mL	5	1	3	2	4	5	1	4	1			
	0.17ug/mL	1	0	0	2	1	3	1	1	0			
	0.06 ug/mL	1	1	1	0	1	1	2	1	1			
dP8	1.5 ug/mL	6	0	1	0	5	4	1	4	1	2.50	3.11	11.83
	0.5 ug/mL	3	1	1	3	5	3	4	5	2			
	0.17ug/mL	7	0	2	1	10	4	4	4	0			
	0.06 ug/mL	5	0	1	1	3	3	3	4	7			
dP9	1.5 ug/mL	1	0	1	0	0	0	2	2	2	0.50	1.00	3.50
	0.5 ug/mL	0	0	1	0	0	1	0	0	0			
	0.17ug/mL	0	3	0	0	0	1	1	0	0			
	0.06 ug/mL	1	0	0	0	0	3	1	1	0			
dP10	1.5 ug/mL	0	0	0	0	0	1	0	0	0	0.25	0.50	1.75
	0.5 ug/mL	0	0	0	0	2	2	0	0	1			
	0.17ug/mL	0	0	0	0	0	0	0	0	0			
	0.06 ug/mL	1	1	0	2	0	1	0	0	0			
dP11	1.5 ug/mL	1	3	2	0	5	4	4	1	1	0.50	0.58	2.23
	0.5 ug/mL	0	3	1	3	1	2	0	0	0			
	0.17ug/mL	4	1	3	0	2	3	0	0	0			
	0.06 ug/mL	1	2	2	1	0	3	0	0	1			
dP12	1.5 ug/mL	0	1	2	3	4	3	3	1	1	1.00	0.82	3.45
	0.5 ug/mL	2	1	4	4	2	5	1	0	1			
	0.17ug/mL	1	0	2	0	0	1	0	2	0			
	0.06 ug/mL	1	0	0	0	7	4	1	2	2			
dP13	1.5 ug/mL	0	1	2	1	0	0	0	0	0	1.00	1.41	5.24
	0.5 ug/mL	2	1	0	2	2	0	1	1	0			
	0.17ug/mL	0	0	0	0	1	1	6	1	1			
	0.06 ug/mL	0	1	3	0	1	0	1	3	3			
dP14	1.5 ug/mL	1	6	2	8	1	3	2	4	2	4.25	3.30	14.16
	0.5 ug/mL	4	6	2	2	3	5	3	7	8			
	0.17ug/mL	3	6	3	5	8	3	2	4	1			
	0.06 ug/mL	2	6	3	1	4	1	1	1	6			
dP15	1.5 ug/mL	7	5	6	4	7	10	6	5	5	4.75	2.87	13.37
	0.5 ug/mL	5	6	1	3	3	2	7	2	1			
	0.17ug/mL	3	4	8	5	5	7	4	0	5			
	0.06 ug/mL	4	7	3	3	7	7	9	6	8			
dP16	1.5 ug/mL	13	18	22	7	23	16	15	11	18	15.67	4.04	27.79
	0.5 ug/mL	8	11	13	11	20	30	11	3	18			
	0.17ug/mL	7	6	8	7	14	9	9	2	11			
	0.06 ug/mL	10	11	11	12	15	15	16	7	56			
dP17	1.5 ug/mL	0	0	1	0	0	3	0	4	0	3.50	3.87	15.12
	0.5 ug/mL	2	0	4	2	1	2	1	1	3			
	0.17ug/mL	0	1	0	3	0	2	1	7	2			
	0.06 ug/mL	1	2	1	3	0	2	2	4	9			
dP18	1.5 ug/mL	0	4	1	2	9	6	5	6	2	2.25	1.71	7.37
	0.5 ug/mL	0	2	1	2	9	9	7	10	4			
	0.17ug/mL	0	4	4	5	9	7	10	5	0			
	0.06 ug/mL	0	1	4	4	3	8	3	5	3			
dC1	1.5 ug/mL	12	15	16	0	27	22	9	22	8	2.00	4.00	14.00
	0.5 ug/mL	13	6	2	0	22	5	5	19	0			
	0.17ug/mL	17	4	0	0	6	9	2	27	0			
	0.06 ug/mL	13	2	2	0	3	4	2	26	0			
dC2	1.5 ug/mL	6	13	6	0	6	15	1	5	1	1.00	0.82	3.45
	0.5 ug/mL	2	16	6	2	9	8	1	5	0			
	0.17ug/mL	3	16	1	0	5	7	1	1	2			
	0.06 ug/mL	1	6	3	1	6	4	0	7	1			
dC3	1.5 ug/mL	4	5	1	2	15	5	4	4	0	0.25	0.50	1.75
	0.5 ug/mL	3	7	0	4	22	11	9	1	1			
	0.17ug/mL	3	6	1	1	16	5	2	2	0			
	0.06 ug/mL	1	5	1	1	11	10	4	3	0			
dC4	1.5 ug/mL	2	14	4	2	7	25	5	9	2	0.75	0.96	3.62
	0.5 ug/mL	3	18	5	3	8	33	3	11	1			
	0.17ug/mL	3	6	6	3	8	24	8	6	0			
	0.06 ug/mL	2	6	38	1	4	15	3	13	0			
dC5	1.5 ug/mL	5	61	0	6	143	45	90	34	4	1.50	1.91	7.24
	0.5 ug/mL	0	53	3	5	105	40	60	41	0			
	0.17ug/mL	5	43	8	6	85	24	26	35	0			
	0.06 ug/mL	5	48	4	4	40	19	22	30	2			
dC6	1.5 ug/mL	5	34	12	2	18	20	4	15	0	1.75	1.26	5.52
	0.5 ug/mL	3	23	2	4	31	19	2	14	3			
	0.17ug/mL	7	15	2	2	14	19	1	11	2			
	0.06 ug/mL	1	4	6	5	15	19	5	15	2			
dC7	1.5 ug/mL	17	14	13	1	30	45	4	22	0	1.25	1.26	5.02
	0.5 ug/mL	14	12	4	0	23	28	7	17	1			
	0.17ug/mL	6	7	0	0	5	10	4	17	1			
	0.06 ug/mL	7	6	0	2	5	4	5	11	3			
dC8	1.5 ug/mL	4	15	12	1	8	14	11	5	0	1.00	1.41	5.24
	0.5 ug/mL	0	4	16	2	8	18	2	5	1			
	0.17ug/mL	1	2	9	0	4	19	3	1	3			
	0.06 ug/mL	1	5	7	0	4	11	8	2	0			
dC9	1.5 ug/mL	0	17	0	0	17	5	7	44	0	0.00	0.00	0.00
	0.5 ug/mL	4	12	0	0	12	9	1	36	0			
	0.17ug/mL	4	9	1	0	6	4	1	17	0			
	0.06 ug/mL	2	8	0	0	7	1	3	12	0			

B

ID.	[ ]	ORF3a	N	Nsp12	Nsp5	S (A)	S (B)	S-RBD	M	PP Neg. Ctrl. $\bar{x}$	σ	$\bar{x}$+3σ
dP1	1.5 ug/mL	0	5	2	3	5	8	4	2	2.83	1.33	6.82
	0.5 ug/mL	0	3	3	1	6	7	8	1			
	0.17ug/mL	0	3	4	0	3	7	0	2			
	0.06 ug/mL	1	5	3	6	2	5	5	1			
dP2	1.5 ug/mL	0	0	3	3	1	6	2	1	1.83	1.17	5.34
	0.5 ug/mL	2	2	1	2	3	0	4	1			
	0.17ug/mL	4	2	2	1	2	3	2	3			
	0.06 ug/mL	2	3	2	1	0	2	1	3			
dP3	1.5 ug/mL	0	4	7	1	1	6	3	1	0.50	0.84	3.01
	0.5 ug/mL	2	2	10	0	4	4	1	3			
	0.17ug/mL	1	1	4	1	5	2	1	1			
	0.06 ug/mL	4	1	3	0	1	3	1	1			
dP4	1.5 ug/mL	12	6	11	5	8	35	6	3	2.17	2.56	9.85
	0.5 ug/mL	15	14	11	7	7	28	0	13			
	0.17ug/mL	4	7	6	10	4	7	1	8			
	0.06 ug/mL	6	12	5	1	4	4	2	1			
dP5	1.5 ug/mL	5	1	3	2	1	1	0	0	4.33	3.56	15.01
	0.5 ug/mL	2	0	0	2	1	3	1	4			
	0.17ug/mL	1	1	2	3	0	1	1	3			
	0.06 ug/mL	2	2	2	1	2	0	2	2			
dP6	1.5 ug/mL	2	3	4	4	3	1	4	1	6.50	2.07	12.72
	0.5 ug/mL	2	4	5	6	3	4	8	2			
	0.17ug/mL	4	5	2	3	0	4	1	6			
	0.06 ug/mL	0	3	2	6	6	3	3	2			
dP7	1.5 ug/mL	3	0	4	1	2	2	4	3	1.67	1.37	5.77
	0.5 ug/mL	5	1	3	2	4	5	1	4			
	0.17ug/mL	1	0	0	2	1	3	1	1			
	0.06 ug/mL	1	1	1	0	1	1	2	1			
dP8	1.5 ug/mL	6	0	1	0	5	4	1	4	2.50	1.05	5.65
	0.5 ug/mL	3	1	1	3	5	3	4	5			
	0.17ug/mL	7	0	2	1	10	4	4	4			
	0.06 ug/mL	5	0	1	1	3	3	3	4			
dP9	1.5 ug/mL	1	0	1	0	0	0	2	2	3.33	2.73	11.53
	0.5 ug/mL	0	0	1	0	0	1	0	0			
	0.17ug/mL	0	3	0	0	0	1	1	0			
	0.06 ug/mL	1	0	0	0	0	3	1	1			
dP10	1.5 ug/mL	0	0	0	0	0	1	0	0	4.50	10.05	34.66
	0.5 ug/mL	0	0	0	0	2	2	0	0			
	0.17ug/mL	0	0	0	0	0	0	0	0			
	0.06 ug/mL	1	1	0	2	0	1	0	0			
dP11	1.5 ug/mL	1	3	2	0	5	4	4	1	1.50	1.87	7.11
	0.5 ug/mL	0	3	1	3	1	2	0	0			
	0.17ug/mL	4	1	3	0	2	3	0	0			
	0.06 ug/mL	1	2	2	1	0	3	0	0			
dP12	1.5 ug/mL	0	1	2	3	4	3	3	1	4.00	7.87	27.62
	0.5 ug/mL	2	1	4	4	2	5	1	0			
	0.17ug/mL	1	0	2	0	0	1	0	2			
	0.06 ug/mL	1	0	0	0	7	4	1	2			
dP13	1.5 ug/mL	0	1	2	1	0	0	0	0	2.00	1.90	7.69
	0.5 ug/mL	2	1	0	2	2	0	1	1			
	0.17ug/mL	0	0	0	0	1	1	6	1			
	0.06 ug/mL	0	1	3	0	1	0	1	3			
dP14	1.5 ug/mL	1	6	2	8	1	3	2	4	7.33	1.75	12.59
	0.5 ug/mL	4	6	2	2	3	5	3	7			
	0.17ug/mL	3	6	3	5	8	3	2	4			
	0.06 ug/mL	2	6	3	1	4	1	1	1			
dP15	1.5 ug/mL	7	5	6	4	7	10	6	5	4.83	1.60	9.64
	0.5 ug/mL	5	6	1	3	3	2	7	2			
	0.17ug/mL	3	4	8	5	5	7	4	0			
	0.06 ug/mL	4	7	3	3	7	7	9	6			
dP16	1.5 ug/mL	13	18	22	7	23	16	15	11	12.00	4.56	25.68
	0.5 ug/mL	8	11	13	11	20	30	11	3			
	0.17ug/mL	7	6	8	7	14	9	9	2			
	0.06 ug/mL	10	11	11	12	15	15	16	7			
dP17	1.5 ug/mL	0	0	1	0	0	3	0	4	2.50	1.22	6.17
	0.5 ug/mL	2	0	4	2	1	2	1	1			
	0.17ug/mL	0	1	0	3	0	2	1	7			
	0.06 ug/mL	1	2	1	3	0	2	2	4			
dP18	1.5 ug/mL	0	4	1	2	9	6	5	6	9.67	5.09	24.92
	0.5 ug/mL	0	2	1	2	9	9	7	10			
	0.17ug/mL	0	4	4	5	9	7	10	5			
	0.06 ug/mL	0	1	4	4	3	8	3	5			
dC1	1.5 ug/mL	12	15	16	0	27	22	9	22	0.67	1.21	4.30
	0.5 ug/mL	13	6	2	0	22	5	5	19			
	0.17ug/mL	17	4	0	0	6	9	2	27			
	0.06 ug/mL	13	2	2	0	3	4	2	26			
dC2	1.5 ug/mL	6	13	6	0	6	15	1	5	1.00	1.55	5.65
	0.5 ug/mL	2	16	6	2	9	8	1	5			
	0.17ug/mL	3	16	1	0	5	7	1	1			
	0.06 ug/mL	1	6	3	1	6	4	0	7			
dC3	1.5 ug/mL	4	5	1	2	15	5	4	4	1.17	1.17	4.67
	0.5 ug/mL	3	7	0	4	22	11	9	1			
	0.17ug/mL	3	6	1	1	16	5	2	2			
	0.06 ug/mL	1	5	1	1	11	10	4	3			
dC4	1.5 ug/mL	2	14	4	2	7	25	5	9	1.17	0.75	3.42
	0.5 ug/mL	3	18	5	3	8	33	3	11			
	0.17ug/mL	3	6	6	3	8	24	8	6			
	0.06 ug/mL	2	6	38	1	4	15	3	13			
dC5	1.5 ug/mL	5	61	0	6	143	45	90	34	3.67	3.14	13.09
	0.5 ug/mL	0	53	3	5	105	40	60	41			
	0.17ug/mL	5	43	8	6	85	24	26	35			
	0.06 ug/mL	5	48	4	4	40	19	22	30			
dC6	1.5 ug/mL	5	34	12	2	18	20	4	15	2.50	1.22	6.17
	0.5 ug/mL	3	23	2	4	31	19	2	14			
	0.17ug/mL	7	15	2	2	14	19	1	11			
	0.06 ug/mL	1	4	6	5	15	19	5	15			
dC7	1.5 ug/mL	17	14	13	1	30	45	4	22	3.83	6.01	21.87
	0.5 ug/mL	14	12	4	0	23	28	7	17			
	0.17ug/mL	6	7	0	0	5	10	4	17			
	0.06 ug/mL	7	6	0	2	5	4	5	11			
dC8	1.5 ug/mL	4	15	12	1	8	14	11	5	0.67	0.82	3.12
	0.5 ug/mL	0	4	16	2	8	18	2	5			
	0.17ug/mL	1	2	9	0	4	19	3	1			
	0.06 ug/mL	1	5	7	0	4	11	8	2			
dC9	1.5 ug/mL	0	17	0	0	17	5	7	44	0.33	0.52	1.88
	0.5 ug/mL	4	12	0	0	12	9	1	36			
	0.17ug/mL	4	9	1	0	6	4	1	17			
	0.06 ug/mL	2	8	0	0	7	1	3	12			

S. Table 4. Positive scoring of SARS-CoV-2 mega peptide pool-induced SFUs in Pre-COVID Era Subjects (IDs dP1-20) and COVID-19-Recovered Subjects (dC1-9) using media control wells **(A)** or irrelevant peptide pools **(B)** as the negative control to establish the background noise. The raw data for establishing the media control (Media Controls), and the irrelevant peptide pool control (PP Neg. Contr.) counts are derived from **S. Table 3**. SARS-Cov2 peptide pools were tested in the specified 4 concentrations [ ] on each PBMC sample. In (A) SFU counts are highlighted that exceed the mean plus 3 SD of the four medium control wells as the cut-off for positivity. In (B) positive SFU counts are highlighted taking the mean and 3 SD of the six irrelevant peptide pool-induced SFU counts as the cut-off for positivity. Note: in pre-COVID donors (dP1-18) the SARS-CoV-2-peptide triggered, allegedly coronavirus cross-reactive responses, are diminished once the background is set to filter out noise created by chance cross-reactivity triggered by the irrelevant control peptide pools.
