# Supplementary material for: Deconvoluting the T Cell Response to SARS-CoV-2: Specificity Versus Chance and Cognate Cross-Reactivity
Source: Front Immunol. 2021 May 28;12:635942. doi: 10.3389/fimmu.2021.635942 (PMC8196231; doi:10.3389/fimmu.2021.635942)
Supplement: Supplementary file 1 [file DataSheet_1.zip › PDF's of All S Material/S Table 5.pdf]

A

ID.	[ ]	ORF3a	N	Nsp12	Nsp5	S (A)	S (B)	S - RBD	M	PP Neg. Ctrl. x̄	σ	x̄+3σ
dC1	1.5 ug/mL	12	15	16	0	27	22	9	22	0.67	1.21	4.30
	0.5 ug/mL	13	6	2	0	22	5	5	19			
	0.17ug/mL	17	4	0	0	6	9	2	27			
	0.06 ug/mL	13	2	2	0	3	4	2	26			
dC2	1.5 ug/mL	6	13	6	0	6	15	1	5	1.00	1.55	5.65
	0.5 ug/mL	2	16	6	2	9	8	1	5			
	0.17ug/mL	3	16	1	0	5	7	1	1			
	0.06 ug/mL	1	6	3	1	6	4	0	7			
dC3	1.5 ug/mL	4	5	1	2	15	5	4	4	1.17	1.17	4.67
	0.5 ug/mL	3	7	0	4	22	11	9	1			
	0.17ug/mL	3	6	1	1	16	5	2	2			
	0.06 ug/mL	1	5	1	1	11	10	4	3			
dC4	1.5 ug/mL	2	14	4	2	7	25	5	9	1.17	0.75	3.42
	0.5 ug/mL	3	18	5	3	8	33	3	11			
	0.17ug/mL	3	6	6	3	8	24	8	6			
	0.06 ug/mL	2	6	38	1	4	15	3	13			
dC5	1.5 ug/mL	5	61	0	6	143	45	90	34	3.67	3.14	13.09
	0.5 ug/mL	0	53	3	5	105	40	60	41			
	0.17ug/mL	5	43	8	6	85	24	26	35			
	0.06 ug/mL	5	48	4	4	40	19	22	30			
dC6	1.5 ug/mL	5	34	12	2	18	20	4	15	2.50	1.22	6.17
	0.5 ug/mL	3	23	2	4	31	19	2	14			
	0.17ug/mL	7	15	2	2	14	19	1	11			
	0.06 ug/mL	1	4	6	5	15	19	5	15			
dC7	1.5 ug/mL	17	14	13	1	30	45	4	22	3.83	6.01	21.87
	0.5 ug/mL	14	12	4	0	23	28	7	17			
	0.17ug/mL	6	7	0	0	5	10	4	17			
	0.06 ug/mL	7	6	0	2	5	4	5	11			
dC8	1.5 ug/mL	4	15	12	1	8	14	11	5	0.67	0.82	3.12
	0.5 ug/mL	0	4	16	2	8	18	2	5			
	0.17ug/mL	1	2	9	0	4	19	3	1			
	0.06 ug/mL	1	5	7	0	4	11	8	2			
dC9	1.5 ug/mL	0	17	0	0	17	5	7	44	0.33	0.52	1.88
	0.5 ug/mL	4	12	0	0	12	9	1	36			
	0.17ug/mL	4	9	1	0	6	4	1	17			
	0.06 ug/mL	2	8	0	0	7	1	3	12			

B

ID.	[ ]	ORF3a	N	Nsp12	Nsp5	S (A)	S (B)	S - RBD	M	PP Neg. Ctrl. x̄	σ	x̄+3σ
dP1	1.5 ug/mL	0	5	2	3	5	8	4	2	2.83	1.33	6.82
	0.5 ug/mL	0	3	3	1	6	7	8	1			
	0.17ug/mL	0	3	4	0	3	7	0	2			
	0.06 ug/mL	1	5	3	6	2	5	5	1			
dP2	1.5 ug/mL	0	0	3	3	1	6	2	1	1.83	1.17	5.34
	0.5 ug/mL	2	2	1	2	3	0	4	1			
	0.17ug/mL	4	2	2	1	2	3	2	3			
	0.06 ug/mL	2	3	2	1	0	2	1	3			
dP3	1.5 ug/mL	0	4	7	1	1	6	3	1	0.50	0.84	3.01
	0.5 ug/mL	2	2	10	0	4	4	1	3			
	0.17ug/mL	1	1	4	1	5	2	1	1			
	0.06 ug/mL	4	1	3	0	1	3	1	1			
dP4	1.5 ug/mL	12	6	11	5	8	35	6	3	2.17	2.56	9.85
	0.5 ug/mL	15	14	11	7	7	28	0	13			
	0.17ug/mL	4	7	6	10	4	7	1	8			
	0.06 ug/mL	6	12	5	1	4	4	2	1			
dP5	1.5 ug/mL	5	1	3	2	1	1	0	0	4.33	3.56	15.01
	0.5 ug/mL	2	0	0	2	1	3	1	4			
	0.17ug/mL	1	1	2	3	0	1	1	3			
	0.06 ug/mL	2	2	2	1	2	0	2	2			
dP6	1.5 ug/mL	2	3	4	4	3	1	4	1	6.50	2.07	12.72
	0.5 ug/mL	2	4	5	6	3	4	8	2			
	0.17ug/mL	4	5	2	3	0	4	1	6			
	0.06 ug/mL	0	3	2	6	6	3	3	2			
dP7	1.5 ug/mL	3	0	4	1	2	2	4	3	1.67	1.37	5.77
	0.5 ug/mL	5	1	3	2	4	5	1	4			
	0.17ug/mL	1	0	0	2	1	3	1	1			
	0.06 ug/mL	1	1	1	0	1	1	2	1			
dP8	1.5 ug/mL	6	0	1	0	5	4	1	4	2.50	1.05	5.65
	0.5 ug/mL	3	1	1	3	5	3	4	5			
	0.17ug/mL	7	0	2	1	10	4	4	4			
	0.06 ug/mL	5	0	1	1	3	3	3	4			
dP9	1.5 ug/mL	1	0	1	0	0	0	2	2	3.33	2.73	11.53
	0.5 ug/mL	0	0	1	0	0	1	0	0			
	0.17ug/mL	0	3	0	0	0	1	1	0			
	0.06 ug/mL	1	0	0	0	0	3	1	1			
dP10	1.5 ug/mL	0	0	0	0	0	1	0	0	4.50	10.05	34.66
	0.5 ug/mL	0	0	0	0	2	2	0	0			
	0.17ug/mL	0	0	0	0	0	0	0	0			
	0.06 ug/mL	1	1	0	2	0	1	0	0			
dP11	1.5 ug/mL	1	3	2	0	5	4	4	1	1.50	1.87	7.11
	0.5 ug/mL	0	3	1	3	1	2	0	0			
	0.17ug/mL	4	1	3	0	2	3	0	0			
	0.06 ug/mL	1	2	2	1	0	3	0	0			
dP12	1.5 ug/mL	0	1	2	3	4	3	3	1	4.00	7.87	27.62
	0.5 ug/mL	2	1	4	4	2	5	1	0			
	0.17ug/mL	1	0	2	0	0	1	0	2			
	0.06 ug/mL	1	0	0	0	7	4	1	2			
dP13	1.5 ug/mL	0	1	2	1	0	0	0	0	2.00	1.90	7.69
	0.5 ug/mL	2	1	0	2	2	0	1	1			
	0.17ug/mL	0	0	0	0	1	1	6	1			
	0.06 ug/mL	0	1	3	0	1	0	1	3			
dP14	1.5 ug/mL	1	6	2	8	1	3	2	4	7.33	1.75	12.59
	0.5 ug/mL	4	6	2	2	3	5	3	7			
	0.17ug/mL	3	6	3	5	8	3	2	4			
	0.06 ug/mL	2	6	3	1	4	1	1	1			
dP15	1.5 ug/mL	7	5	6	4	7	10	6	5	4.83	1.60	9.64
	0.5 ug/mL	5	6	1	3	3	2	7	2			
	0.17ug/mL	3	4	8	5	5	7	4	0			
	0.06 ug/mL	4	7	3	3	7	7	9	6			
dP16	1.5 ug/mL	13	18	22	7	23	16	15	11	12.00	4.56	25.68
	0.5 ug/mL	8	11	13	11	20	30	11	3			
	0.17ug/mL	7	6	8	7	14	9	9	2			
	0.06 ug/mL	10	11	11	12	15	15	16	7			
dP17	1.5 ug/mL	0	0	1	0	0	3	0	4	2.50	1.22	6.17
	0.5 ug/mL	2	0	4	2	1	2	1	1			
	0.17ug/mL	0	1	0	3	0	2	1	7			
	0.06 ug/mL	1	2	1	3	0	2	2	4			
dP18	1.5 ug/mL	0	4	1	2	9	6	5	6	9.67	5.09	24.92
	0.5 ug/mL	0	2	1	2	9	9	7	10			
	0.17ug/mL	0	4	4	5	9	7	10	5			
	0.06 ug/mL	0	1	4	4	3	8	3	5			

S. TABLE 5. Affinity analysis of SFU counts triggered by SARS-CoV-2 peptides in PBMC of donors who recovered from PCR-verified SARS- CoV-2 infection **(A)** and in pre-Covid Era subjects **(B).** Affinity levels are color-coded. Red: high affinity, defined as four consecutive peptide dilutions eliciting a positive recall response with SFU counts exceeding 3 SD of the negative peptide pool-based background (PP Neg. Contr.), as specified for each PBMC sample on the right. Orange: intermediate affinity, defined as three consecutive peptide dilutions eliciting a positive recall response. Yellow: low affinity, with only the two highest peptide concentrations eliciting positive SFU counts. Beige: only the highest concentration of peptide is positive.
