# Supplementary material for: Deconvoluting the T Cell Response to SARS-CoV-2: Specificity Versus Chance and Cognate Cross-Reactivity
Source: Front Immunol. 2021 May 28;12:635942. doi: 10.3389/fimmu.2021.635942 (PMC8196231; doi:10.3389/fimmu.2021.635942)
Supplement: Supplementary file 1 [file DataSheet_1.zip › PDF's of All S Material/S Table 6.pdf]

A

ID.	[ ]	BARF1	BMLF1	BMRF1	BRLF1	BZLF1	EBNA-LP	EBNA1	EBNA2	EBNA3a	EBNA3b	EBNA3c	GP350/340	LMP1	LMP2	PP Neg. Ctrl. x̄	σ	x̄+3σ
dC1	1.5 ug/mL	2	0	8	19	22	15	9	31	20	18	13	21	1	19	0.67	1.21	4.30
	0.5 ug/mL	1	1	5	10	21	11	17	21	12	11	12	35	0	27			
	0.17ug/mL	1	2	0	3	8	14	14	21	13	9	12	32	6	19			
	0.06 ug/mL	0	1	5	2	13	8	7	20	11	9	9	16	10	23			
dC2	1.5 ug/mL	2	2	1	1	11	13	9	11	6	1	50	13	5	12	1.00	1.55	5.65
	0.5 ug/mL	2	2	1	0	6	16	7	8	4	4	26	3	4	14			
	0.17ug/mL	1	2	1	0	3	2	4	10	2	0	10	2	2	10			
	0.06 ug/mL	0	1	3	1	2	1	1	8	3	1	3	2	0	7			
dC3	1.5 ug/mL	0	0	2	40	18	41	55	2	15	11	57	7	3	13	1.17	1.17	4.67
	0.5 ug/mL	1	3	0	24	12	40	45	1	34	4	46	9	3	18			
	0.17ug/mL	0	0	2	23	15	23	39	0	26	4	44	2	4	7			
	0.06 ug/mL	1	1	1	28	2	20	32	1	17	3	23	0	2	2			
dC4	1.5 ug/mL	1	1	4	51	10	58	41	7	58	5	7	20	0	18	1.17	0.75	3.42
	0.5 ug/mL	2	1	1	34	10	96	46	8	65	4	7	19	4	11			
	0.17ug/mL	0	0	0	36	7	73	40	5	70	2	6	11	3	16			
	0.06 ug/mL	1	4	0	19	2	45	44	7	44	1	6	4	2	13			
dC5	1.5 ug/mL	7	4	9	1	4	10	17	9	33	8	49	5	11	9	3.67	3.14	13.09
	0.5 ug/mL	0	4	13	5	5	5	16	19	30	9	20	6	7	3			
	0.17ug/mL	1	0	11	3	4	6	10	9	8	4	18	7	4	8			
	0.06 ug/mL	0	2	6	2	3	2	5	10	7	7	8	1	5	1			
dC6	1.5 ug/mL	17	4	25	6	5	9	4	3	8	6	10	29	4	4	2.50	1.22	6.17
	0.5 ug/mL	6	6	16	3	5	2	8	6	4	5	4	38	6	4			
	0.17ug/mL	4	8	0	2	5	1	16	4	5	3	9	8	9	4			
	0.06 ug/mL	15	9	2	0	1	2	7	3	4	8	4	3	5	2			
dC7	1.5 ug/mL	10	6	22	4	27	14	9	7	6	6	72	52	2	10	3.83	6.01	21.87
	0.5 ug/mL	7	3	6	1	8	3	5	2	1	1	61	19	3	15			
	0.17ug/mL	1	4	0	1	2	7	2	2	1	4	32	4	4	11			
	0.06 ug/mL	1	1	1	2	3	1	1	6	1	3	17	7	0	5			
dC8	1.5 ug/mL	3	10	4	7	134	9	8	22	8	23	4	0	0	23	0.67	0.82	3.12
	0.5 ug/mL	5	6	9	7	103	7	12	22	1	7	1	0	4	12			
	0.17ug/mL	3	6	11	6	130	4	7	12	1	10	3	6	0	5			
	0.06 ug/mL	6	3	6	8	99	3	2	22	3	4	6	0	1	6			
dC9	1.5 ug/mL	1	23	48	10	7	12	89	25	4	5	75	3	0	9	0.33	0.52	1.88
	0.5 ug/mL	1	2	40	2	6	5	49	17	3	2	47	0	0	8			
	0.17ug/mL	0	3	47	7	2	7	36	13	1	7	35	0	2	10			
	0.06 ug/mL	0	2	50	5	4	8	17	13	0	0	15	0	1	8			

B

ID.	[ ]	BARF1	BMLF1	BMRF1	BRLF1	BZLF1	EBNA-LP	EBNA1	EBNA2	EBNA3a	EBNA3b	EBNA3c	GP350/340	LMP1	LMP2	PP Neg. Ctrl. x̄	σ	x̄+3σ
dP1	1.5 ug/mL	19	2	2	5	5	30	7	13	30	5	7	9	2	3	2.83	1.33	6.82
	0.5 ug/mL	5	2	3	0	0	12	6	8	18	1	4	4	2	5			
	0.17ug/mL	2	2	3	4	1	2	4	6	13	2	3	6	6	2			
	0.06 ug/mL	0	2	6	4	0	3	5	4	6	2	3	2	2	2			
dP2	1.5 ug/mL	3	1	1	0	2	18	4	2	10	3	2	3	1	6	1.83	1.17	5.34
	0.5 ug/mL	0	2	0	1	0	10	4	4	10	2	4	2	1	2			
	0.17ug/mL	1	0	0	2	0	7	2	4	9	2	4	1	0	1			
	0.06 ug/mL	0	2	1	1	0	8	2	3	4	0	2	1	1	0			
dP3	1.5 ug/mL	5	2	1	88	4	7	1	1	6	3	1	7	3	2	0.50	0.84	3.01
	0.5 ug/mL	12	1	2	49	2	10	0	4	4	1	3	6	4	2			
	0.17ug/mL	3	1	1	25	1	4	1	5	2	1	1	0	14	1			
	0.06 ug/mL	2	1	3	14	1	3	0	1	3	1	1	5	6	0			
dP4	1.5 ug/mL	13	14	7	5	6	11	5	8	35	6	3	20	8	4	2.17	2.56	9.85
	0.5 ug/mL	11	7	9	14	14	11	7	7	28	0	13	14	32	19			
	0.17ug/mL	10	4	3	8	7	6	10	4	7	1	8	3	29	13			
	0.06 ug/mL	7	4	3	8	12	5	1	4	4	2	1	2	3	3			
dP5	1.5 ug/mL	5	3	4	6	2	22	13	18	20	9	14	7	1	6	4.33	3.56	15.01
	0.5 ug/mL	2	4	3	5	0	5	11	30	7	6	8	1	2	9			
	0.17ug/mL	2	3	18	5	1	5	16	29	6	5	4	4	1	5			
	0.06 ug/mL	1	4	3	3	1	1	3	23	5	7	5	2	0	1			
dP6	1.5 ug/mL	6	3	6	3	0	6	4	1	1	6	7	2	2	3	6.50	2.07	12.72
	0.5 ug/mL	6	4	7	3	2	7	6	2	6	10	3	5	4	5			
	0.17ug/mL	6	5	11	11	4	2	2	2	5	3	5	5	2	2			
	0.06 ug/mL	5	6	8	9	0	1	4	3	4	0	3	0	6	1			
dP7	1.5 ug/mL	1	3	3	8	19	58	60	19	77	10	42	12	1	8	1.67	1.37	5.77
	0.5 ug/mL	0	2	3	12	14	59	46	18	73	2	27	2	2	5			
	0.17ug/mL	0	1	2	8	9	36	44	10	42	4	10	6	2	2			
	0.06 ug/mL	1	6	3	5	4	37	36	9	37	1	5	2	0	9			
dP8	1.5 ug/mL	7	1	6	10	10	25	50	14	30	19	17	6	5	24	2.50	1.05	5.65
	0.5 ug/mL	11	8	5	11	4	17	36	11	20	12	24	7	7	22			
	0.17ug/mL	5	5	6	11	5	6	22	11	13	14	11	6	5	23			
	0.06 ug/mL	4	4	13	8	7	9	18	8	9	14	21	2	4	12			
dP9	1.5 ug/mL	1	6	42	5	4	37	21	2	42	0	29	2	0	19	3.33	2.73	11.53
	0.5 ug/mL	4	5	45	3	11	81	22	1	26	1	38	0	1	8			
	0.17ug/mL	2	0	37	0	7	68	36	3	26	1	26	3	0	14			
	0.06 ug/mL	1	1	37	0	5	26	15	2	11	0	10	0	0	5			
dP10	1.5 ug/mL	1	2	1	96	11	230	29	110	222	93	18	12	2	16	4.50	10.05	34.66
	0.5 ug/mL	2	3	4	67	1	217	22	91	196	64	12	1	2	6			
	0.17ug/mL	1	2	9	24	5	210	21	76	184	60	7	0	3	4			
	0.06 ug/mL	0	3	2	12	6	171	19	99	157	40	17	2	3	0			
dP11	1.5 ug/mL	4	52	6	8	143	87	5	10	13	7	9	10	4	10	1.50	1.87	7.11
	0.5 ug/mL	5	52	3	4	129	33	10	5	2	4	7	5	1	3			
	0.17ug/mL	1	38	3	1	150	8	6	11	10	5	2	1	1	8			
	0.06 ug/mL	1	20	3	0	114	3	13	8	3	2	2	0	0	3			
dP12	1.5 ug/mL	0	1	5	7	45	292	83	15	282	6	6	22	2	4	4.00	7.87	27.62
	0.5 ug/mL	0	1	4	1	30	274	76	15	249	8	17	16	0	4			
	0.17ug/mL	2	2	4	0	14	250	64	9	254	8	3	7	1	8			
	0.06 ug/mL	0	2	2	0	1	191	44	2	183	3	6	3	0	4			
dP13	1.5 ug/mL	2	3	1	1	4	33	3	21	19	7	49	10	5	3	2.00	1.90	7.69
	0.5 ug/mL	3	1	2	2	4	19	2	33	9	17	38	7	4	2			
	0.17ug/mL	1	2	2	1	3	9	0	23	20	20	29	2	0	0			
	0.06 ug/mL	3	2	2	2	2	5	1	18	10	7	12	1	3	0			
dP14	1.5 ug/mL	12	2	109	108	6	87	21	70	25	101	153	7	3	9	7.33	1.75	12.59
	0.5 ug/mL	8	4	103	82	5	48	16	66	9	103	98	4	3	2			
	0.17ug/mL	2	3	91	47	7	27	48	59	8	110	88	6	2	4			
	0.06 ug/mL	5	10	88	24	2	13	16	51	7	76	51	3	4	4			
dP15	1.5 ug/mL	5	9	6	16	14	28	16	46	59	23	17	18	6	10	4.83	1.60	9.64
	0.5 ug/mL	4	3	5	13	5	8	15	47	20	11	23	13	2	9			
	0.17ug/mL	5	3	5	5	4	2	16	65	7	12	22	7	3	3			
	0.06 ug/mL	0	3	2	5	3	7	18	40	7	15	9	6	2	5			
dP16	1.5 ug/mL	29	12	11	27	183	94	53	32	15	41	82	22	21	23	12.00	4.56	25.68
	0.5 ug/mL	18	15	9	14	87	84	46	34	10	15	90	13	13	11			
	0.17ug/mL	13	18	8	7	40	34	50	24	4	8	49	22	5	14			
	0.06 ug/mL	8	13	5	10	27	23	48	20	1	11	41	8	10	9			
dP17	1.5 ug/mL	6	4	11	1	70	14	18	1	37	1	3	13	3	4	2.50	1.22	6.17
	0.5 ug/mL	2	2	5	2	65	1	11	5	19	21	4	9	4	8			
	0.17ug/mL	1	1	6	0	43	8	8	2	9	1	7	2	0	2			
	0.06 ug/mL	5	4	6	2	19	6	1	1	2	1	0	3	3	0			
dP18	1.5 ug/mL	4	8	7	3	0	2	9	6	18	1	30	0	4	1	9.67	5.09	24.92
	0.5 ug/mL	11	6	16	3	3	0	7	11	12	3	9	0	2	1			
	0.17ug/mL	5	4	8	8	1	3	3	7	1	0	8	0	0	0			
	0.06 ug/mL	5	2	5	9	0	0	0	3	0	1	2	0	1	0			

S. TABLE 6. Affinity analysis of SFU counts triggered by EBV mega peptide pools in PBMC of donors recovered from PCR-verified SARS-CoV-2- infection **(A)** and in Pre-COVID Era Subjects **(B)**. The EBV peptides are closer defined in S. Table 2. Except for the peptides tested, the assay was performed and analyzed as for Table 2.
