# Supplementary material for: Deconvoluting the T Cell Response to SARS-CoV-2: Specificity Versus Chance and Cognate Cross-Reactivity
Source: Front Immunol. 2021 May 28;12:635942. doi: 10.3389/fimmu.2021.635942 (PMC8196231; doi:10.3389/fimmu.2021.635942)
Supplement: Supplementary file 1 [file DataSheet_1.zip › PDF's of All S Material/S Table 7.pdf]

A

ID.	[ ]	HKU1 S (A)	HKU1 S (B)	229E S (A)	229E S (B)	NL63 S (A)	NL63 S (B)	OC43 S (A)	OC43 S (B)	PP Neg. Ctrl. x̄	σ	x̄+3σ
dC1	1.5 ug/mL	3	8	0	11	1	9	4	25	0.67	1.21	4.30
	0.5 ug/mL	5	11	1	5	1	13	2	11			
	0.17ug/mL	10	8	2	11	1	8	5	4			
	0.06 ug/mL	2	6	3	3	1	5	3	9			
dC2	1.5 ug/mL	1	17	3	11	2	28	6	5	1.00	1.55	5.65
	0.5 ug/mL	5	8	2	13	4	24	6	9			
	0.17ug/mL	4	6	9	6	3	20	8	10			
	0.06 ug/mL	8	6	0	9	3	18	6	7			
dC3	1.5 ug/mL	4	7	0	4	3	8	2	19	1.17	1.17	4.67
	0.5 ug/mL	7	7	1	3	5	14	10	12			
	0.17ug/mL	3	5	1	5	22	9	20	13			
	0.06 ug/mL	3	0	0	2	2	16	19	4			
dC4	1.5 ug/mL	3	25	10	14	6	21	7	8	1.17	0.75	3.42
	0.5 ug/mL	0	9	6	14	2	15	0	12			
	0.17ug/mL	0	6	3	9	4	12	2	4			
	0.06 ug/mL	5	7	3	4	13	27	3	10			
dC5	1.5 ug/mL	1	10	3	21	5	21	3	11	3.67	3.14	13.09
	0.5 ug/mL	8	9	7	10	2	16	7	7			
	0.17ug/mL	2	3	3	9	4	13	3	9			
	0.06 ug/mL	0	1	2	1	1	13	4	7			
dC6	1.5 ug/mL	0	34	1	13	3	22	12	14	2.50	1.22	6.17
	0.5 ug/mL	4	21	2	12	3	27	4	8			
	0.17ug/mL	3	24	2	21	3	18	14	6			
	0.06 ug/mL	3	5	4	15	10	12	6	6			
dC7	1.5 ug/mL	10	3	0	27	3	25	13	6	3.83	6.01	21.87
	0.5 ug/mL	4	5	3	14	5	24	14	10			
	0.17ug/mL	7	2	4	13	4	17	11	4			
	0.06 ug/mL	3	1	3	5	0	8	3	2			
dC8	1.5 ug/mL	7	4	11	9	6	13	2	3	0.67	0.82	3.12
	0.5 ug/mL	12	3	12	4	9	8	0	9			
	0.17ug/mL	10	7	8	10	2	5	3	1			
	0.06 ug/mL	3	1	5	1	3	5	1	7			
dC9	1.5 ug/mL	0	3	6	9	0	13	2	1	0.33	0.52	1.88
	0.5 ug/mL	2	4	4	2	0	5	0	1			
	0.17ug/mL	3	0	8	4	0	0	0	1			
	0.06 ug/mL	1	0	8	2	0	2	0	0			

B

ID.	[ ]	HKU1 S (A)	HKU1 S (B)	229E S (A)	229E S (B)	NL63 S (A)	NL63 S (B)	OC43 S (A)	OC43 S (B)	PP Neg. Ctrl. x̄	σ	x̄+3σ
dP1	1.5 ug/mL	19	7	8	6	13	14	6	28	2.83	1.33	6.82
	0.5 ug/mL	18	4	0	6	7	11	11	25			
	0.17ug/mL	22	5	3	9	6	11	5	13			
	0.06 ug/mL	13	10	3	9	5	10	3	20			
dP2	1.5 ug/mL	1	2	1	1	1	6	4	8	1.83	1.17	5.34
	0.5 ug/mL	2	0	4	2	0	3	3	0			
	0.17ug/mL	1	3	1	3	2	1	2	1			
	0.06 ug/mL	3	0	2	0	2	8	2	1			
dP3	1.5 ug/mL	7	3	2	0	1	1	1	2	0.50	0.84	3.01
	0.5 ug/mL	6	4	2	2	2	1	0	2			
	0.17ug/mL	0	14	1	1	2	2	0	0			
	0.06 ug/mL	5	6	0	4	2	0	0	1			
dP4	1.5 ug/mL	20	8	4	12	3	0	1	1	2.17	2.56	9.85
	0.5 ug/mL	14	32	19	15	3	0	1	1			
	0.17ug/mL	3	29	13	4	0	1	1	6			
	0.06 ug/mL	2	3	3	6	0	1	0	2			
dP5	1.5 ug/mL	1	6	9	7	2	11	6	2	4.33	3.56	15.01
	0.5 ug/mL	7	8	2	4	5	5	3	4			
	0.17ug/mL	4	8	3	1	1	6	10	1			
	0.06 ug/mL	1	2	2	3	5	4	12	1			
dP6	1.5 ug/mL	1	4	5	7	4	6	7	6	6.50	2.07	12.72
	0.5 ug/mL	2	4	6	7	6	4	0	4			
	0.17ug/mL	3	2	4	5	2	8	4	3			
	0.06 ug/mL	2	6	1	5	1	4	3	0			
dP7	1.5 ug/mL	10	10	5	16	1	15	6	11	1.67	1.37	5.77
	0.5 ug/mL	13	0	10	13	3	20	7	6			
	0.17ug/mL	8	4	3	5	5	6	8	4			
	0.06 ug/mL	3	2	3	4	4	5	4	5			
dP8	1.5 ug/mL	4	10	15	11	11	23	4	1	2.50	1.05	5.65
	0.5 ug/mL	3	10	15	21	6	14	3	8			
	0.17ug/mL	5	5	14	6	9	10	7	3			
	0.06 ug/mL	4	3	12	6	8	6	2	4			
dP9	1.5 ug/mL	9	7	3	4	2	7	1	5	3.33	2.73	11.53
	0.5 ug/mL	11	0	2	3	0	4	0	3			
	0.17ug/mL	5	1	1	1	1	4	3	4			
	0.06 ug/mL	1	1	0	1	5	25	5	4			
dP10	1.5 ug/mL	2	0	2	2	1	6	2	7	4.50	10.05	34.66
	0.5 ug/mL	1	3	0	1	0	4	5	4			
	0.17ug/mL	2	0	0	0	0	2	3	1			
	0.06 ug/mL	1	1	0	0	1	1	2	2			
dP11	1.5 ug/mL	9	28	2	16	6	39	13	22	1.50	1.87	7.11
	0.5 ug/mL	9	11	3	12	6	50	8	14			
	0.17ug/mL	6	19	0	15	4	18	5	26			
	0.06 ug/mL	10	10	0	9	3	17	9	10			
dP12	1.5 ug/mL	9	11	7	27	9	22	15	15	4.00	7.87	27.62
	0.5 ug/mL	5	15	2	15	4	30	17	12			
	0.17ug/mL	8	7	6	9	3	17	10	7			
	0.06 ug/mL	7	4	2	7	3	8	5	1			
dP13	1.5 ug/mL	10	15	13	9	0	18	3	4	2.00	1.90	7.69
	0.5 ug/mL	9	7	5	11	3	12	0	8			
	0.17ug/mL	15	13	9	7	1	13	3	6			
	0.06 ug/mL	6	7	12	6	1	8	1	0			
dP14	1.5 ug/mL	12	11	7	28	8	19	15	10	7.33	1.75	12.59
	0.5 ug/mL	8	13	8	30	7	20	23	7			
	0.17ug/mL	13	8	9	24	9	14	20	4			
	0.06 ug/mL	4	5	5	6	5	11	14	2			
dP15	1.5 ug/mL	7	12	15	30	3	14	20	10	4.83	1.60	9.64
	0.5 ug/mL	10	14	10	11	9	18	10	7			
	0.17ug/mL	5	8	10	17	10	11	11	6			
	0.06 ug/mL	2	6	3	15	10	13	10	8			
dP16	1.5 ug/mL	21	42	25	63	103	91	70	67	12.00	4.56	25.68
	0.5 ug/mL	18	28	18	34	68	53	56	48			
	0.17ug/mL	12	25	21	26	59	70	43	70			
	0.06 ug/mL	13	25	16	25	44	69	56	70			
dP17	1.5 ug/mL	2	4	5	5	13	27	2	10	2.50	1.22	6.17
	0.5 ug/mL	2	1	15	7	6	10	4	5			
	0.17ug/mL	3	1	1	8	4	19	4	4			
	0.06 ug/mL	3	2	4	4	5	10	23	6			
dP18	1.5 ug/mL	8	12	18	33	9	19	4	12	9.67	5.09	24.92
	0.5 ug/mL	8	8	13	14	6	22	14	16			
	0.17ug/mL	7	10	13	26	12	8	3	11			
	0.06 ug/mL	13	2	10	10	8	6	5	2			

S. TABLE 7. T cell recall responses to Spike proteins of the four Common Cold Coronaviruses, 229E, NL63, OC43, and HKU1, each represented due to size in two peptide pools. Each peptide pools has been tested in the specified four concentrations in a standard IFN-γ ELISPOT assay. SFU counts exceeding 3 SD of the mean of the negative peptide pool control (PP. Neg. Contr.) shown on the left for each subject, are highlighted according to T cell affinity levels, as specified in Table 2.
